# Structural insights into full-length human fascin1: a target for cancer treatment

**DOI:** 10.1107/S2053230X25005254

**Published:** 2025-06-27

**Authors:** Lucía Giraldo-Ruiz, Isabel Quereda-Moraleda, Alice Grieco, Javier Ruiz-Sanz, Irene Luque, Jose Manuel Martin-Garcia

**Affiliations:** a Department of Physical Chemistry, Institute of Biotechnology and Excellence Unit in Chemistry Applied to Biomedicine and Environment, Faculty of Sciences University of Granada Granada Spain; b Department of Crystallography and Structural Biology Institute of Physical Chemistry Blas Cabrera, Spanish National Research Council (CSIC) Madrid Spain; University of Essex, United Kingdom

**Keywords:** human fascin1, cancer, metastasis, dynamics, plasticity, actin-binding proteins, X-ray crystallography

## Abstract

Fascin1 proteins are a family of globular proteins with actin-bundling activity that cross-link actin filaments together, allowing the formation of actin-rich structures involved in cell migration and adhesion, such as filopodia, invadopodia, stress fibers, micro-spikes and podocytes. The overexpression of human fascin1 has been linked to tumor progression in most human cancers, particularly during the epithelial–mesenchymal transition, making it a promising biomarker for cancer metastasis and a major target for the development of novel cancer therapies. X-ray crystallography has been instrumental in human fascin1-inhibition research since it provides detailed insights into the structure of the protein and its interactions with small-molecule inhibitors. This technique has allowed the characterization of a range of molecular conformations in which the protein naturally exists. However, human fascin1 has never been fully modeled until now. To the best of our knowledge, this study presents the first full-length structure of human fascin1 in which both copies are fully resolved. Comparison of this structure with the available wild-type and complexed structures provides new insights into the conformational plasticity of fascin1 that will facilitate subsequent studies on human fascin1 in the context of drug design for cancer-related therapies.

## Abstract 2

This study presents the first fully resolved crystal structure of human fascin1, revealing its conformational plasticity and key interdomain interactions critical for actin bundling and cancer metastasis. These findings provide a structural foundation for the development of allosteric inhibitors and pave the way for time-resolved crystallographic studies to explore fascin1 dynamics in metastasis and drug targeting.

## 1. Introduction

**Figure 1:**
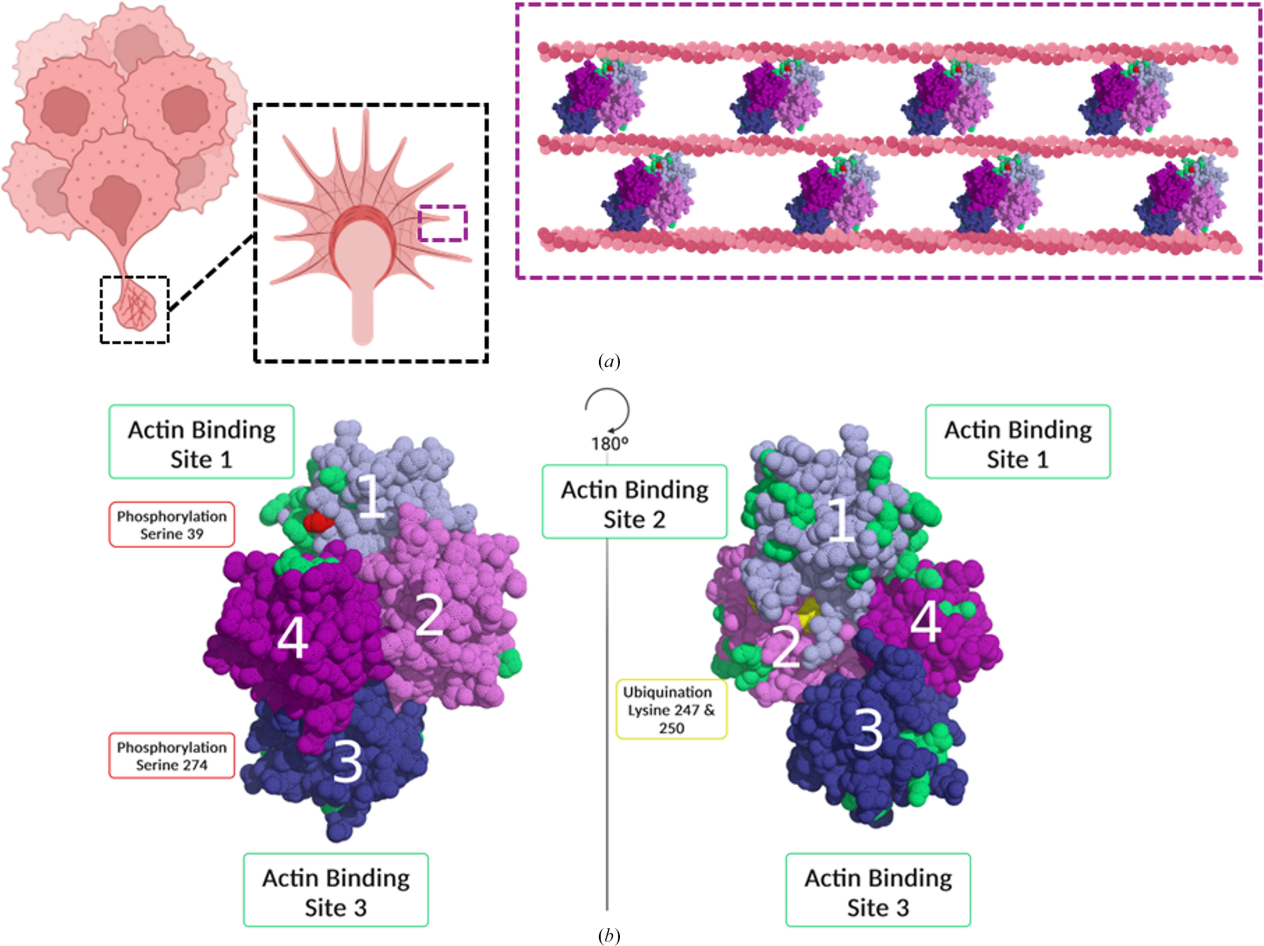
Fascin1-mediated actin bundling in tumor-cell protrusions. (*a*) Schematic representation of tumor cells forming membrane protrusions during tumor progression. The black dashed box highlights an individual tumor cell, where these protrusions emerge. The purple dashed box provides a closer view of the actin filaments, showing how fascin1 cross-links them into tightly packed bundles via its actin-binding sites. (*b*) Structural representation of fascin1, shown in two orientations rotated 180° along the displayed axis. The four β-trefoil domains (labeled 1–4) are shown, with actin-binding sites (green) located at distinct regions. Key post-translational modifications involved in fascin1 regulation are also indicated: phosphorylation sites (red) and ubiquitination sites (yellow).

Fascin1 (*FSCN1* gene) is an actin-bundling protein that organizes actin filaments into compact parallel arrays (Machesky & Li, 2010 ▸; Adams, 2004 ▸; Fig. 1 ▸*a*), facilitating the formation of dynamic cellular structures (Elkhatib *et al.*, 2014 ▸) that are critical for the generation of plasma-membrane protrusions (filopodia and invadopodia) necessary for cell adhesion, motility and invasion (Machesky & Li, 2010 ▸; Adams, 2004 ▸). Fascin1 plays a pivotal role in the development of metastasis, which is the primary cause of mortality in cancer patients (Sarantelli *et al.*, 2023 ▸). While it is absent or present at minimal concentrations in most adult human cells, fascin1 is overexpressed in almost all metastatic cancers, including breast, prostate, ovarian, lung, liver and pancreatic cancers, among others. In addition, a number of studies have confirmed fascin1 as a novel and universal biomarker for aggressive human cancers with poor prognosis and low survival rates (Hashimoto *et al.*, 2011 ▸). Fascin1 is also an attractive target for the development of novel antitumor therapies aimed at specifically blocking cell migration and reducing the development of metastasis in aggressive cancers that do not respond to traditional antiproliferative treatments. Several studies have been conducted to develop fascin1 inhibitors as novel antimetastatic therapies (Chen *et al.*, 2010 ▸; Huang *et al.*, 2015 ▸, 2018 ▸; Alburquerque-González *et al.*, 2020 ▸, 2021 ▸, 2024 ▸; Burgess *et al.*, 2024 ▸; Asensi-Cantó *et al.*, 2023 ▸; Francis *et al.*, 2019 ▸; Riahi *et al.*, 2019 ▸), such as migrastatin and its macroketone analogs, which have been shown to reduce metastatic tumor-cell migration, invasion and metastasis (Chen *et al.*, 2010 ▸; Huang *et al.*, 2015 ▸, 2018 ▸; Alburquerque-González *et al.*, 2020 ▸, 2021 ▸, 2024 ▸; Burgess *et al.*, 2024 ▸; Asensi-Cantó *et al.*, 2023 ▸; Francis *et al.*, 2019 ▸; Riahi *et al.*, 2019 ▸.

The cross-linking activity of fascin1 is closely related to its structure and dynamic properties (Hashimoto *et al.*, 2011 ▸; Sedeh *et al.*, 2010 ▸). Fascin1 is a compact 55 kDa protein composed of a pseudo-tetrahedral arrangement of four β-trefoil-like domains (F1–F4) organized into two semi-independent lobes (F1–F2 and F3–F4) arranged in a V-shape at an angle of approximately 56° (Hashimoto *et al.*, 2011 ▸; Sedeh *et al.*, 2010 ▸; Fig. 1 ▸*b*). This unique structural arrangement facilitates coupling between the domains, thereby influencing their actin-bundling activity (Hashimoto *et al.*, 2011 ▸; Sedeh *et al.*, 2010 ▸). Although high-resolution structural data for the fascin1–actin complex remain limited, recent studies have provided valuable insights into its architecture and actin-binding mechanisms (Gong *et al.*, 2025 ▸; Chung *et al.*, 2022 ▸). Chung *et al.* (2022 ▸) employed electron microscopy to reveal the three-dimensional structure of the fascin1–actin complex, identifying that the N-terminal region, which includes actin-binding site 2 (ABS2), plays a crucial role in actin bundling. The C-terminal region was found to facilitate fascin1 dimerization, suggesting a key structural feature for its function (Chung *et al.*, 2022 ▸). Similarly, Gong *et al.* (2025 ▸) used cryo-electron microscopy and tomography to explore the role of fascin1 in actin bundling, demonstrating that its structural plasticity enables it to accommodate various inter-filament orientations, thereby bridging mismatches between the helical symmetry of F-actin and the hexagonal packing of the actin bundles (Gong *et al.*, 2025 ▸). Two major binding sites have been identified (Yang *et al.*, 2013 ▸): site 1, consisting of the N- and C-terminal regions and the cleft between the F1 and F4 domains, and site 2, located on the opposite side of the molecule and consisting of amino acids from the F1 and F2 domains. These binding sites are shown in Fig. 1 ▸(*b*). In addition to these, a third actin-binding site in the F3 domain has been proposed, further expanding our understanding of the interaction of fascin1 with actin filaments (Sedeh *et al.*, 2010 ▸; Chen *et al.*, 2010 ▸; Fig. 1 ▸*b*). Despite the lack of high-resolution structural data for the complete fascin1–actin complex, these findings collectively enhance our understanding of the dynamic architecture and functional binding sites that facilitate the roles of fascin1 in actin bundling and filament cross-linking. Fascin1 interacts with actin in a cooperative manner (Yamakita *et al.*, 1996 ▸) and it has been proposed that sites 1 and 2, located at the top of the molecule, bind to two actin monomers in the same fiber, while site 3, located at the bottom, interacts with another filament (Sedeh *et al.*, 2010 ▸; Chen *et al.*, 2010 ▸; Yang *et al.*, 2013 ▸; Fig. 1 ▸*c*). The actin-bundling activity of fascin1 is tightly regulated by post-translational modifications (Nan-Li *et al.*, 2022 ▸). In particular, phosphorylation by protein kinase Cα of a critical serine residue (Ser39) located in the region of site 1, which inhibits the bundling activity of fascin1 without affecting its localization to filopodial tips (Elkhatib *et al.*, 2014 ▸; Chen *et al.*, 2010 ▸; Sedeh *et al.*, 2010 ▸), influences the formation of structures (invadopodia) critical for cancer-cell invasion and is crucial for cellular dynamics and morphology.

Fascin1 is a protein characterized by high conformational flexibility, a property essential for its actin-bundling function. It has been proposed that the binding of fascin1 to the first monomer of actin stabilizes a conformation that is competent for interaction with additional actin monomers, generating the cooperative effect observed experimentally. The ‘active conformation’ of fascin1 is associated with a proper spatial alignment of the major actin-binding sites, enabling efficient cross-linking of actin filaments and the formation of cellular protrusions such as filopodia. This state typically corresponds to the dephosphorylated wild-type protein and is functionally linked to enhanced cell migration and invasion. In contrast, a structurally distinct ‘inactive conformation’ has been observed in crystallographic studies of both wild-type and mutant fascin1 proteins, including the phosphomimetic mutant S39D and other variants that impair actin interaction (Yang *et al.*, 2013 ▸). Interestingly, although these mutations are distributed across different regions of the protein, the associated conformational changes are mainly localized around site 2 in the F2 domain, suggesting an allosteric mechanism of conformational stabilization (Yang *et al.*, 2013 ▸). The structural differences between the ‘active’ and ‘inactive’ conformations of fascin1 are subtle, limited to variations in loop and β-strand geometry that disrupt actin-binding competence without drastically altering the overall fold of the protein.

In addition to post-translational modifications and mutations, ligand binding can induce more pronounced structural rearrangements in fascin1. Crystal structures of fascin1 in complex with the small-molecule inhibitors NP-G2-029 (PDB entry 6b0t; Huang *et al.*, 2018 ▸) and BDP13176 (PDB entry 6ioz; Francis *et al.*, 2019 ▸) reveal a dramatic rotation of approximately 35° in domain F1 upon ligand binding. This reorientation creates a hydrophobic cleft between domains 1 and 2, and results in a distortion of both actin-binding sites, thereby abrogating bundling activity (Huang *et al.*, 2018 ▸). Functionally, this conformational shift correlates with inhibition of actin-filament bundling and reduced tumor-cell migration, invasion and metastasis (Huang *et al.*, 2018 ▸; Jansen *et al.*, 2011 ▸). Moreover, crystallographic structures of several BDP13176 variants illustrate the capacity of this region to accommodate diverse ligand geometries and sizes, highlighting the structural plasticity of fascin1 and its potential for allo­steric modulation (Francis *et al.*, 2019 ▸). These findings underscore the importance of conformational dynamics in regulating fascin1 activity and support its relevance as a therapeutic target for the development of novel antimetastatic agents.

Here, we present the crystal structure of full-length human fascin1 in its high-activity or active conformation solved to 2.2 Å resolution. This active state is characterized by dephosphorylation at Ser39 and optimal alignment of the two actin-binding sites located in domains 1 and 3, which together enable efficient filament bundling. We analyzed its packing, conformational flexibility and cooperative domain inter­actions to gain further insight into the structural and functional dynamics of fascin1. Our study provides new insights into the mechanistic basis of its actin-bundling activity, directly dependent on the dephosphorylated, active form, and highlights its potential as a therapeutic target for inhibitor design. We have also developed preliminary crystallization conditions for time-resolved serial femtosecond crystallography (TR-SFX). Ongoing TR-SFX experiments could unravel the molecular mechanisms underlying the role of fascin1 in cancer metastasis and offer valuable insights for the design of novel antimetastatic therapies targeting this protein.

## 2. Materials and methods

### 2.1. Materials

For protein expression, Luria–Bertani (LB) medium, ampicillin, isopropyl β-d-1-thiogalactopyranoside (IPTG), Tris, NaCl, EDTA, DTT and glutathione were obtained from Sigma–Aldrich, Darmstadt, Germany, *Escherichia coli* BL21 (DE3) cells were acquired from Novagen, Darmstadt, Germany, protease-inhibitor tablets were purchased from Roche, DNase I was purchased from Sigma–Aldrich, Darmstadt, Germany and Pre-Scission Protease (PSS) was obtained from Cytiva, Uppsala, Sweden.

For protein purification, Glutathione Sepharose 4B resin and an ÄKTA go system were obtained from Cytiva, Uppsala, Sweden. The HiLoad 16/600 Superdex 200 prep-grade column was from GE Healthcare, Barcelona, Spain. Protein fractions were analyzed by sodium dodecyl sulfate–polyacrylamide gel electrophoresis (SDS–PAGE). Protein concentration was carried out using 30 kDa concentrators from Millipore, Madrid, Spain.

For protein crystallization, HEPES and polyethylene glycol 4000 were purchased from Sigma–Aldrich, Darmstadt, Germany, isopropyl alcohol was purchased from Quimipur, Madrid, Spain, glycerol was purchased from Fisher Chemical, Madrid, Spain, liquid N_2_ was supplied by Linde Gas, Valencia, Spain, 96-well crystallization plates were purchased from Sigma–Aldrich, Darmstadt, Germany and the Milli-Q Type I Ultrapure Water Production System was obtained from Merck Millipore, Darmstadt, Germany.

### 2.2. Expression of human fascin1

Human fascin1 fused to glutathione *S*-transferase (GST) was expressed according to the protocol established by Huang *et al.* (2018 ▸) with some modifications. A starter of 5 ml LB medium supplemented with ampicillin was inoculated with 5 µl of an *E. coli* BL21 (DE3) culture previously transformed with pGEX-6P-2 plasmid and grown overnight at 37°C. The next day, the starter was transferred to 1 l LB medium supplemented with ampicillin and incubated at 37°C until the optical density at 600 nm (OD_600_) reached a value of between 0.6 and 0.8. The culture was then induced with 0.5 m*M* IPTG and incubated overnight at 25°C. The cells were subsequently harvested by centrifugation at 3300*g* for 15 min. The cell pellets were resuspended in 10 ml PBS buffer (140 mm NaCl, 2.7 m*M* KCl, 10 m*M* Na_2_HPO_4_, 1.8 m*M* KH_2_PO_4_ pH 7.4), flash-frozen with liquid N_2_ and stored at −80°C.

### 2.3. Purification of human fascin1

Purification of GST-human fascin1 was carried out following the protocol previously reported by Sedeh *et al.* (2010 ▸) with some variations. Frozen PBS-resuspended cells were thawed and one protease-inhibitor tablet and 5 µl DNase were added. The cells were disrupted by sonication using three cycles of 2 min each, alternating 2 s on and 2 s off, with 2 min resting on ice. The lysate was clarified by ultracentrifugation at 803 000*g* at 4°C for 30 min. The supernatant was then added to Glutathione Sepharose 4B resin previously equilibrated with PBS buffer. The fusion protein was eluted with 10 m*M* glutathione, 50 m*M* Tris pH 8.0 and dialyzed overnight against 50 m*M* Tris, 150 m*M* NaCl, 1 m*M* EDTA, 1 m*M* DTT pH 7.0. Human fascin1 was liberated from the GST tag by a 4 h cleavage with Pre-Scission Protease (PSS, Cytiva) and subsequent Glutathione Sepharose affinity chromatography. The purity of all protein fractions throughout the purification process was monitored using SDS–PAGE (Supplementary Figs. S1*a* and S1*b*). Purified human fascin1 was finally dialyzed extensively against 20 m*M* HEPES pH 7.5, 100 m*M* NaCl, flash-frozen with liquid N_2_ and stored at −80°C.

An additional purification step was required to obtain the protein microcrystals (see Section 2.5). This was performed using size-exclusion chromatography on a HiLoad 16/600 Superdex 200 prep-grade column with an elution buffer composed of 20 m*M* HEPES pH 7.5, 100 m*M* NaCl (Supplementary Fig. S1*c*). The pure protein was concentrated to 10 mg ml^−1^, flash-frozen with liquid N_2_ and stored at −80°C.

### 2.4. Crystallization of human fascin1

Crystallization of the human fascin1 protein was carried out using the sitting-drop vapor-diffusion method in 24-well plates. In outline, 1 µl protein solution at 15–20 mg ml^−1^ was mixed with 1 µl precipitant solution composed of 0.1 *M* HEPES pH 7.5, 20% polyethylene glycol (PEG) 4000, 2% 2-propanol. Protein droplets were equilibrated against 500 µl precipitant solution in the reservoir at 20°C. Prior to data collection, the crystals were soaked in a cryoprotectant solution consisting of the precipitant solution supplemented with 20% glycerol, looped, cryocooled in liquid N_2_, stored in a dry-shipper and shipped to the synchrotron-radiation facility for data collection.

### 2.5. Data collection and structure determination

X-ray diffraction data collection was performed on the BL-13 XALOC beamline at the ALBA synchrotron-radiation source, Barcelona, Spain using a wavelength of 0.98 Å and a PILATUS X 6M detector. Indexing of the collected data sets was performed with *XDS* (Kabsch, 2010 ▸), and scaling and merging were performed with *AIMLESS* (Evans & Murshudov, 2013 ▸) from the *CCP*4 suite (Agirre *et al.*, 2023 ▸). A 5% fraction of reflections was included in the generated *R*_free_ set. Phasing was performed by employing molecular replacement with *MOLREP* (Vagin & Teplyakov, 2010 ▸) using the crystal structure of human fascin1 with PDB code 3llp (Chen *et al.*, 2010 ▸) as the search model. The obtained model was refined with *REFMAC*5 (Murshudov *et al.*, 2011 ▸), with iterative cycles of automated refinement and manual rebuilding in *Coot* (Emsley *et al.*, 2010 ▸). Noncrystallographic symmetry (NCS) restraints were applied, and translation–libration–screw (TLS) refinement was implemented from the outset, with each β-trefoil domain defined as an independent TLS group to account for structural modularity and domain flexibility. This approach was used to improve the geometry while minimizing overfitting. Water molecules were automatically upgraded after each refinement cycle. The final refined structure was validated using the wwwPDB Validation Service and submitted to the PDB for deposition as PDB entry 9fn6. All data-collection and refinement statistics are summarized in Table 1 ▸. All figures showing the protein structure presented in this work were generated with *PyMOL* (version 3.1.3; Schrödinger).

### 2.6. Microcrystallization assays

Microcrystallization of human fascin1 was performed by optimizing the crystallization conditions used to obtain the large crystals described in Section 2.4. A range of crystallization conditions were assayed by varying the concentration and molecular weight of PEG, the protein concentration and the protein:precipitant ratio, as well as the incorporation of additives along with the use of seeding. Crystallization was carried out using the batch method, where the protein solution is quickly added to and mixed with the precipitant solution. Seeding was employed to increase the crystal density and size homogeneity while reducing the crystallization time. The seeds were obtained by fragmenting one-day-old large crystals (100–200 µm) grown at 18°C using the hanging-drop vapor-diffusion method under the aforementioned crystallization conditions. The best needle-shaped microcrystals, measuring approximately 25–30 µm in their longest dimension, were obtained overnight using a precipitant solution consisting of 0.1 *M* HEPES pH 7.5, 30% PEG 4000, 2% 2-propanol, 50 m*M* Li_2_SO_4_, with 1 µl of seed stock added per 100 µl of precipitant. Crystallization was performed at a protein concentration of 10 mg ml^−1^, using a 1:3 protein:precipitant ratio and incubating at 18°C. The seed stock was prepared by crushing large crystals (100–200 µm) grown over 24 h at 18°C by hanging-drop vapor diffusion using a similar precipitant solution (0.1 *M* HEPES pH 7.5, 20% PEG 4000, 2% 2-propanol, 50 m*M* Li_2_SO_4_) with a protein concentration of 10 mg ml^−1^ and a 1:3 protein:precipitant ratio. All crystal samples were analyzed using high-resolution optical microscopy.

## 3. Results

### 3.1. Crystal structure of the full-length human fascin1 protein

**Figure 2:**
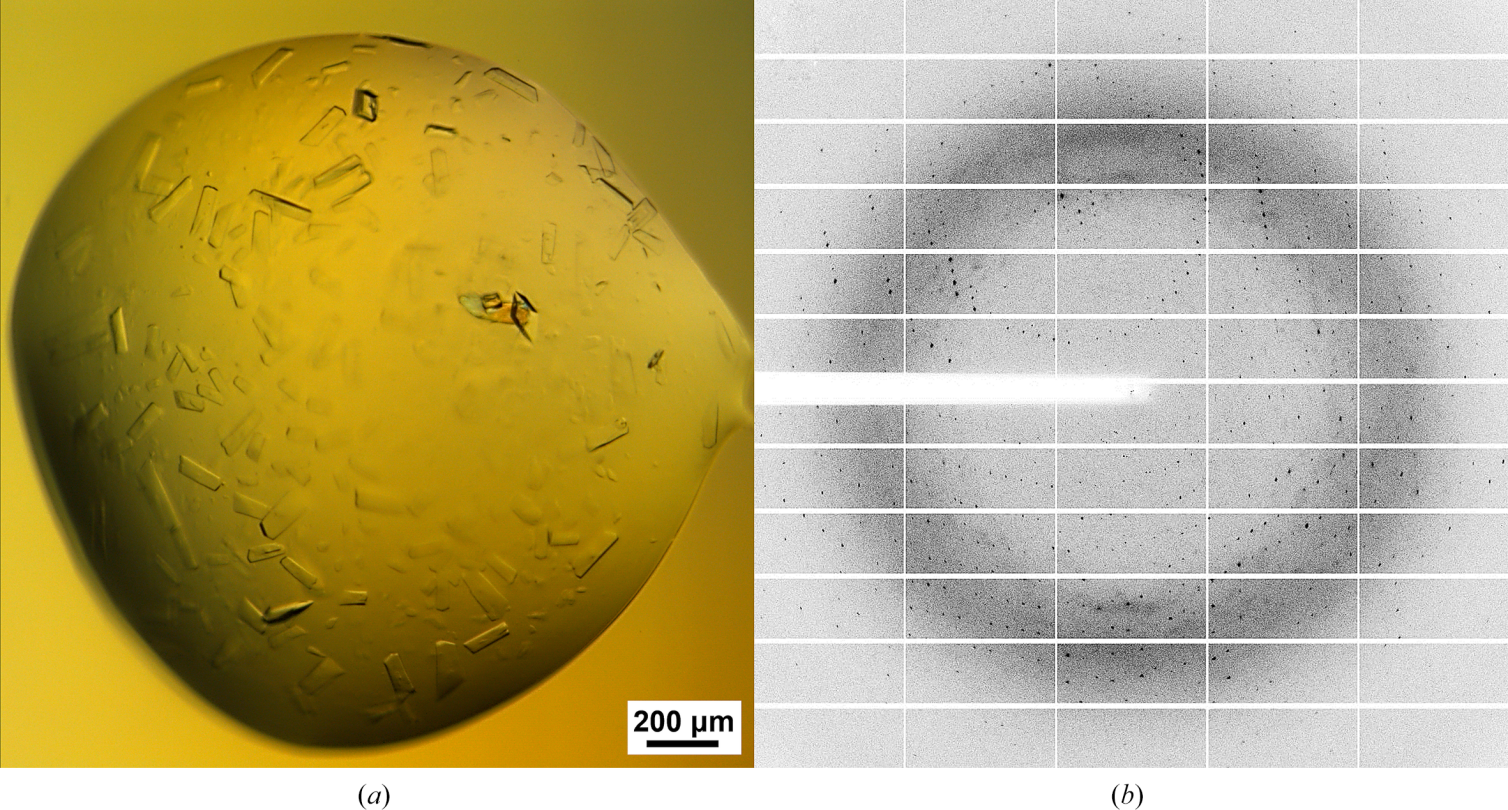
Crystallization and diffraction of the human fascin1 protein. (*a*) Large human fascin1 crystals obtained by the sitting-drop vapor-diffusion method. (*b*) A representative diffraction pattern from the crystals obtained in (*a*).

Pure protein from the last purification step was subjected to crystallization. The best human fascin1 crystals (100 µm; Fig. 2 ▸*a*) were obtained in 0.1 *M* HEPES pH 7.0, 20% PEG 4000, 2% propanol using the hanging-drop vapor-diffusion method. The crystals belonged to space group *I*121, with unit-cell parameters *a* = 114.57, *b* = 70.80, *c* = 122.21 Å, α = 90.00, β = 92.78, γ = 90.00°. The crystals were observed to diffract beyond 2.0 Å resolution (Fig. 2 ▸*b*). A total of 2400 images were collected and successfully indexed, integrated and merged. Two molecules of human fascin1, chains *A* and *B*, were found in the asymmetric unit, yielding a Matthews coefficient (*V*_M_) of 2.23 A^3^ Da^−1^ and a solvent content of 44.98%. The structure of human fascin1 was solved by molecular replacement using PDB entry 3llp (Chen *et al.*, 2010 ▸), from which the solvent molecules had been previously removed, as a search model. The structure was refined to a final resolution of 2.2 Å, with *R*_work_ and *R*_free_ values of 23.4% and 30.4%, respectively. The final data-collection and refinement statistics are given in Table 1 ▸.

**Figure 3:**
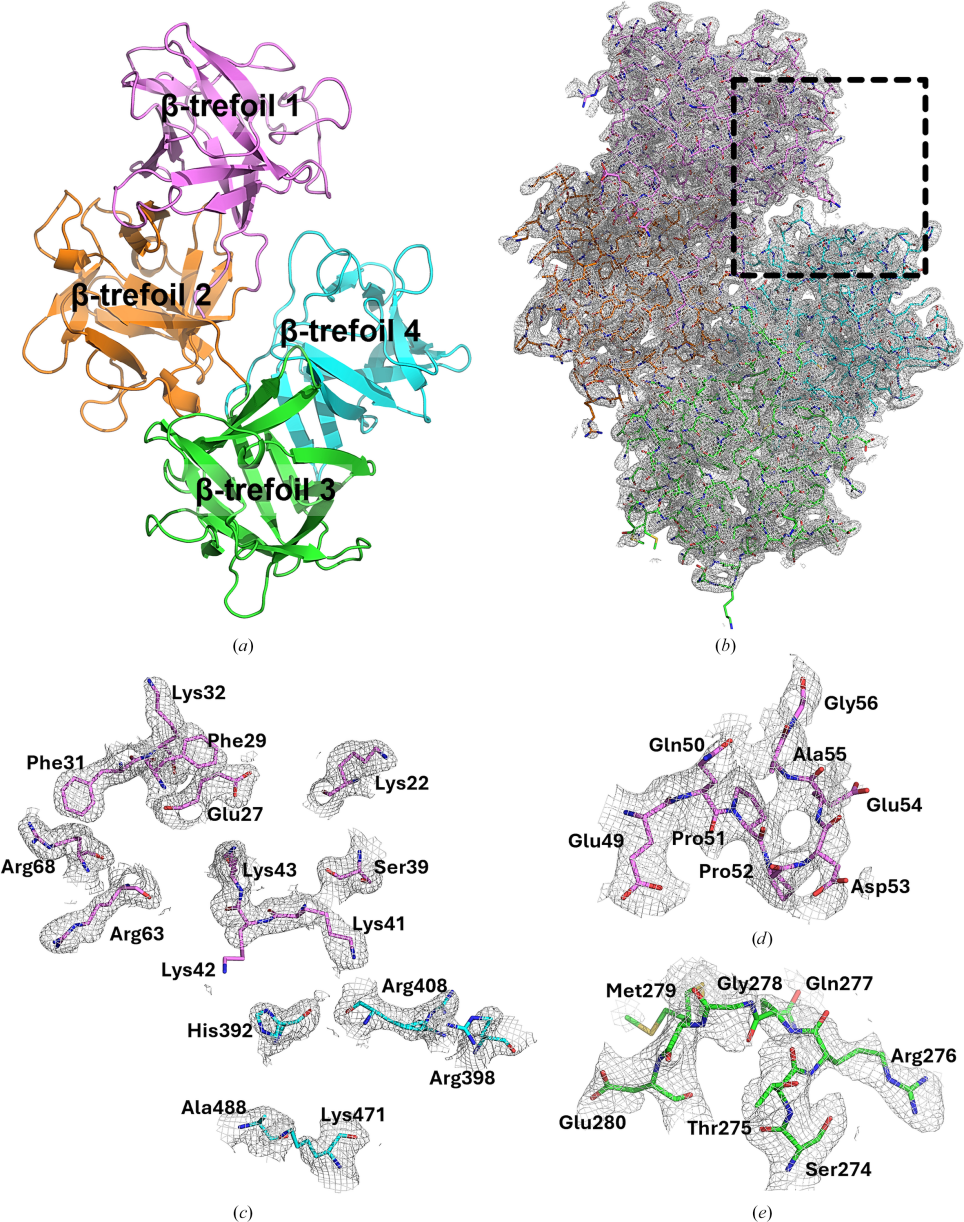
Crystal structure of the full-length human fascin1 protein. (*a*) Cartoon representation of molecule *A* in the asymmetric unit. β-Trefoil domain 1 is shown in magenta, β-trefoil domain 2 is shown in orange, β-trefoil domain 3 is shown in green and β-trefoil domain 4 is shown in blue. (*b*) 2*mF*_o_ − *DF*_c_ electron-density map contoured at 1σ of the whole protein. The dashed black box highlights the ABS1 region. (*c*) 2*mF*_o_ − *DF*_c_ electron-density map contoured at 1σ of the residues comprising ABS1 of chain *A* highlighted in (*b*). (*d*) 2*mF*_o_ − *DF*_c_ electron-density map contoured at 1σ of residues 49–56 in β-trefoil domain 1. (*e*) 2*mF*_o_ − *DF*_c_ electron-density map contoured at 1σ of residues 274–280 in β-trefoil domain 3.

All 493 residues of human fascin1 were manually inspected, and loop regions lacking initial density were built *de novo* in *Coot* (Emsley *et al.*, 2010 ▸) based on the electron-density maps. The model spans from the N-terminus to the C-terminus without interruptions, except for the first seven N-terminal residues, which could not be modeled due to the absence of interpretable density. The resulting structure shows the typical fascin1 fold containing four β-trefoil domains (residues 8–139 for β-trefoil domain 1, residues 140–260 for β-trefoil domain 2, residues 261–381 for β-trefoil domain 3 and residues 382–493 for β-trefoil domain 4). Fig. 3 ▸(*a*) illustrates the molecule of human fascin1 corresponding to chain *A* in the asymmetric unit. The stereochemistry of the structure was excellent, with root-mean-square deviations (r.m.s.d.s) from the ideal of 0.012 Å for bond lengths and less than 1.823° for bond angles (Table 1 ▸). The Ramachandran diagram places most residues within the favored (91%) and allowed (7%) regions, and only 2% of the residues fall into the outlier region (Table 1 ▸). The resulting experimental maps revealed the presence of 308 water molecules. The high quality of our structure can also be assessed from the 2*mF*_o_ − *DF*_c_ electron-density map of the entire protein (Fig. 3 ▸*b*), as well as those for the ABS1 region (Fig. 3 ▸*c* for chain *A* and Supplementary Fig. S2*a* for chain *B*) and the regions of the protein that were not modeled in previous crystal structures (Figs. 3 ▸*d* and 3 ▸*e* for chain *A* and Supplementary Figs. S2*b* and S2*c* for chain *B*).

### 3.2. Comparison of the human fascin1 structure with related structures

Currently, three structures of human fascin1 in its wild-type form have been deposited in the PDB [PDB entries 1dfc (Sedeh *et al.*, 2010 ▸), 3llp (Chen *et al.*, 2010 ▸) and 3p53 (Jansen *et al.*, 2011 ▸)]. However, due to the exceptionally high flexibility reported in the literature for this protein, several regions in β-trefoil domains 1 and 3 were often missing in one of the two molecules within the asymmetric unit. The crystal structure presented in this study is, to the best of our knowledge, the first full-length structure of human fascin1 in which all residues of the two molecules of the asymmetric unit have been modeled fully. As an example, Figs. 3 ▸(*d*) and 3 ▸(*e*) highlight two highly flexible regions, residues 49–56 in β-trefoil domain 1 and residues 274–280 in β-trefoil domain 3, which have been fully modeled for the first time in the two molecules of the asymmetric unit of our structure.

**Figure 4:**
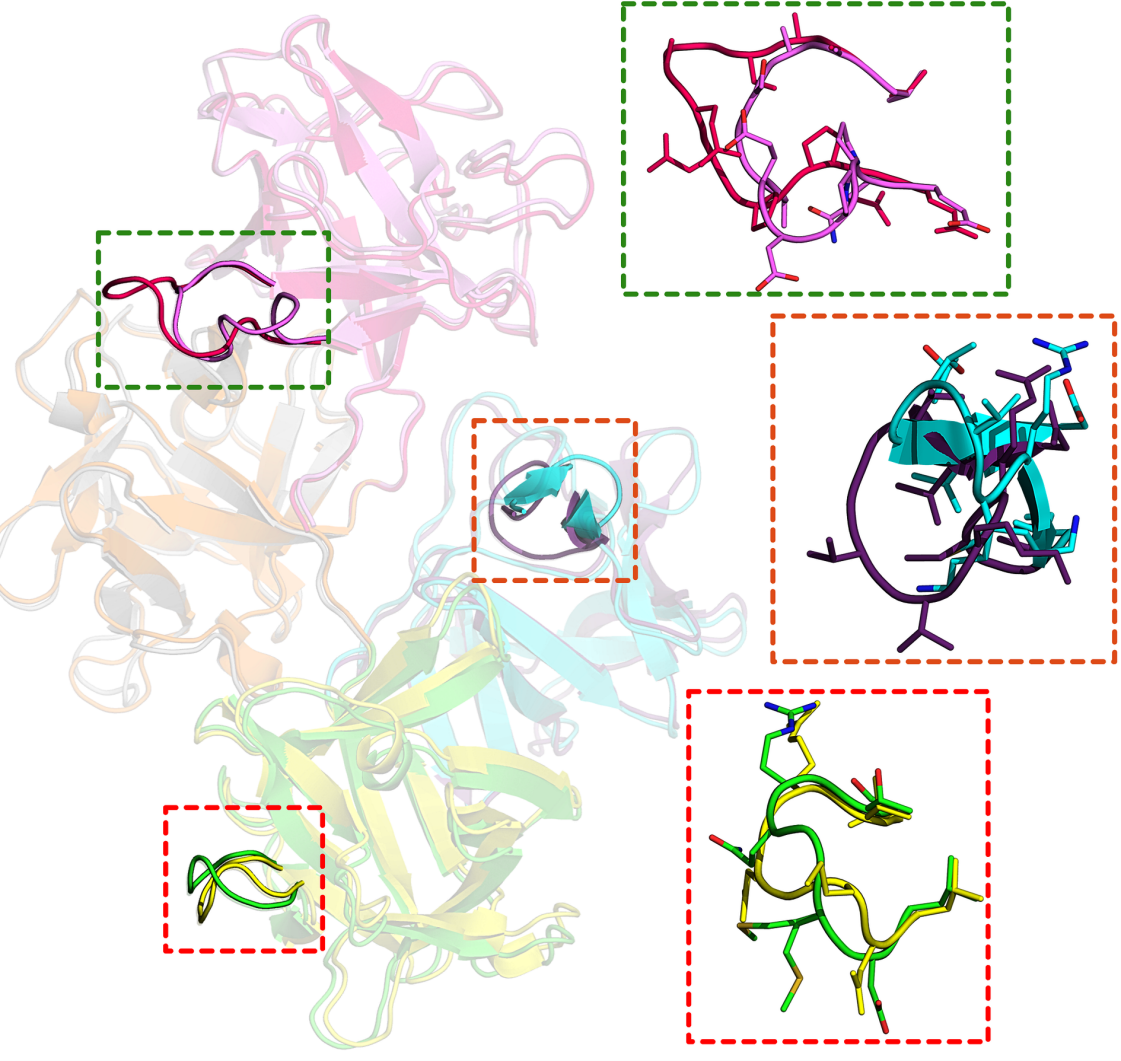
Structural comparison. Superimposition of the two molecules *A* (β-trefoil domain 1 in magenta, β-trefoil domain 2 in orange, β-trefoil domain 3 in green and β-trefoil domain 4 in blue) and *B* (β-trefoil domain 1 in pink, β-trefoil domain 2 in gray, β-trefoil domain 3 in yellow and β-trefoil domain 4 in purple) in the asymmetric unit of the human fascin1 structure presented in this study. Regions 1, 2 and 3, consisting of residues 49–60 in β-trefoil domain 1, 274–281 in β-trefoil domain 3 and 395–405 in β-trefoil domain 4, respectively, are enclosed in dashed boxes. A closer view of each region is also provided.

To further investigate the flexibility of human fascin1, we carried out a structural alignment of the two molecules in the asymmetric unit and observed that even though the r.m.s.d. value indicates that the two molecules are similar (1.13 Å), a visual inspection of the superimposition shows that there are significant differences between them (Fig. 4 ▸). These differences are mostly found in loop regions, especially in the regions comprising residues 49–60 in β-trefoil domain 1, residues 274–281 in β-trefoil domain 3 and residues 395–405 in β-trefoil domain 4, which have been modeled in a different conformation in each molecule of the asymmetric unit (Fig. 4 ▸). It is important to mention that the electron density around the residues of these two regions is clearly visible and the backbone was well modeled. In addition to this, we also compared the four individual β-trefoil domains with each other for the two molecules found in the asymmetric unit (Supplementary Table S1). Supplementary Fig. S3 shows a comparison of the individual β-trefoil domains with each other and Supplementary Table S1 collects the overall r.m.s.d.s from this comparison. Interestingly, β-trefoil domains 1 and 3, which have been reported to be somewhat interconnected (Lamb & Tootle, 2020 ▸; Huang *et al.*, 2018 ▸; Sedeh *et al.*, 2010 ▸; Chen *et al.*, 2010 ▸; Jansen *et al.*, 2011 ▸), show the lowest r.m.s.d., indicating that these two domains of human fascin1 are structurally similar compared with domains 2 and 4.

Further evaluation of the human fascin1 structure was carried out by comparing it with previously reported crystal structures in its free form [PDB entries 1dfc (Sedeh *et al.*, 2010 ▸), 3llp (Chen *et al.*, 2010 ▸) and 3p53 (Jansen *et al.*, 2011 ▸)]. Overall, all human fascin1 structures aligned well with each other, with r.m.s.d values for all atoms of between 0.65 and 1.70 Å and an average value of 1.23 Å. Supplementary Fig. S4 shows the superimposition of our structure with all free human fascin1 structures. As expected, the larger structural differences are mainly found in the loop regions and solvent-exposed areas, as reflected by the higher r.m.s.d. values. However, there are several regions in which significant conformational changes are observed (residues 49–59, 156–162, 274–280, 298–305 and 397–404). Supplementary Fig. S4 also shows a closer view of these flexible regions. This highlights the importance of our full-length structure in obtaining a complete picture of the protein folding, which was missing in other related structures.

**Figure 5:**
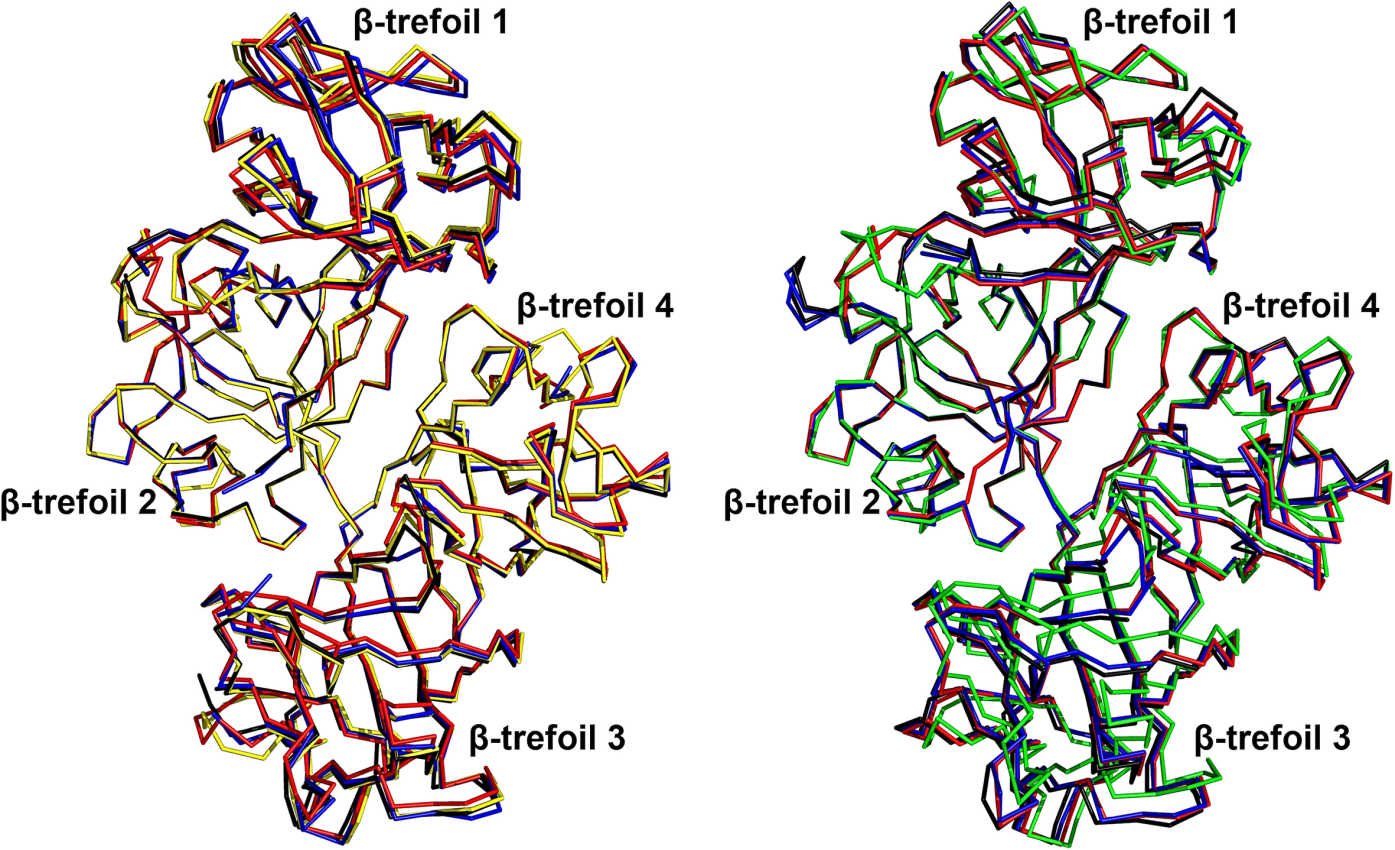
Structural variability within crystal structures of the free human fascin1 protein. Ribbon representations of molecule *A* (left panel) and molecule *B* (right panel) from various crystal structures of human fascin1. Structures shown include wild-type fascin1 from the present study (yellow for molecule *A*, green for molecule *B*), wild-type fascin1 from PDB entry 3llp (Chen *et al.*, 2010 ▸; black), wild-type fascin1 from PDB entry 3p53 (Jansen *et al.*, 2011 ▸; red) and the inactive S39D mutant from PDB entry 4gov (Yang *et al.*, 2013 ▸; blue). To highlight conformational differences and domain flexibility, all structures were superposed based on β-trefoil domain 2. The comparisons reveal variations in domain orientations that underline the structural plasticity of fascin1 and its functional regulation.

In addition to these local differences within the individual β-trefoil domains, high variability is also observed when comparing their relative orientations. This is illustrated in Fig. 5 ▸, where the different structures available for free fascin1 are compared. In this figure, the F2 domains of all structures have been superposed in order to highlight the ‘breathing’ dynamics of the protein. As can be observed, the orientation of β-trefoil domain 1 and most of β-trefoil domain 3 varies significantly, even though the internal structures of the domains remain mostly invariable, as described before. Interestingly, while molecule *A* in our structure lies within the conformational dynamics previously observed in structures of free wild-type fascin1 [PDB entries 3llp (Chen *et al.*, 2010 ▸) and 3p53 (Jansen *et al.*, 2011 ▸)] and the inactive S39D mutant (PDB entry 4gov; Yang *et al.*, 2013 ▸), molecule *B* captures larger conformational departures, clearly illustrating the high plasticity of free fascin1 (Fig. 5 ▸).

### 3.3. Analysis of salt-bridge interactions in human fascin1

**Figure 6:**
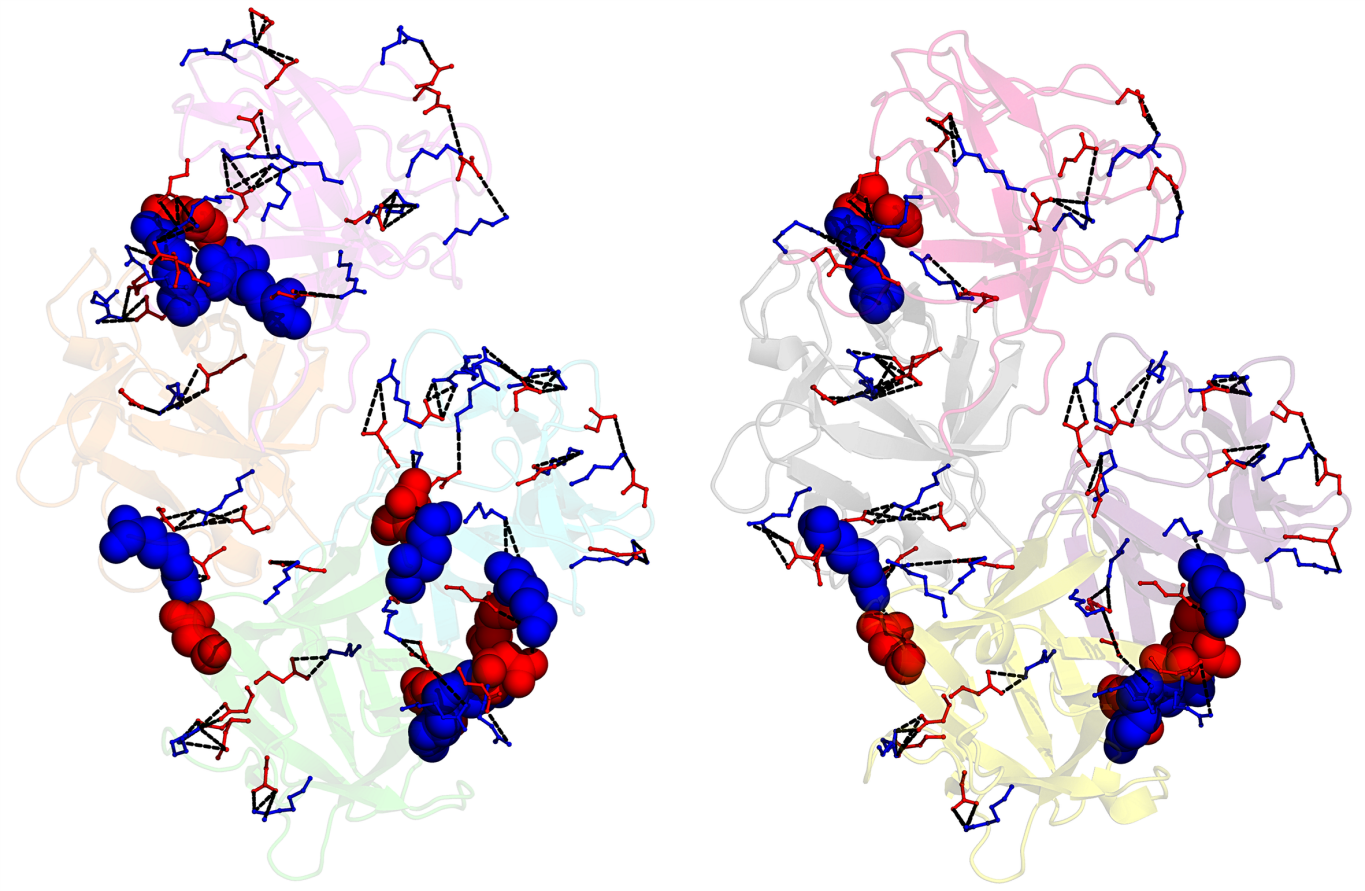
Salt-bridge interactions in human fascin1. Ball-and-stick representation of all salt bridges found in molecules *A* (left panel) and *B* (right panel) in the asymmetric unit of the structure reported in this study. Positive residues (Arg and Lys) are shown in blue and negative residues (Glu and Asp) are shown in red. Salt bridges are shown as black dashed lines. Residues involved in interdomain interactions are shown as spheres. Fascin1 molecules are shown in a cartoon representation using the same color code as in Fig. 4 ▸.

Fascin1 is a highly charged protein with a total of 134 (27.1%) charged residues across the protein, including 77 that are positively charged (15.6%) and 57 that are negatively charged (11.6%). Using *PyMOL* with a minimum distance cutoff of 2.2 Å and a maximum distance cutoff of 5 Å, we have identified all salt bridges across chains *A* and *B* (Supplementary Table S3). The number of salt bridges differs slightly between the two molecules in the asymmetric unit, with molecule *A* having 103 and molecule *B* having 101 (Table 2 ▸). The most significant differences between the two molecules lie in the distribution of their interactions throughout the protein. While slight variations are observed across β-trefoil domains 3 and 4, β-trefoil domains 1 and 2 show the greatest differences in salt-bridge distribution (Table 2 ▸ and Fig. 6 ▸). More importantly, among all salt bridges, we have found several β-trefoil interdomain interactions which could be responsible for the stability of the protein (Table 2 ▸ and Fig. 6 ▸). In molecule *A*, β-trefoil domain 1 is connected to β-trefoil domain 2 via Asp97, which forms salt bridges with Arg185 and Arg224 in β-trefoil domain 2 (Table 2 ▸). The link between β-trefoil domain 2 and β-trefoil domain 3 is mediated by an interaction between Arg167 and Asp290 (Table 2 ▸). Likewise, β-trefoil domain 3 is connected to β-trefoil domain 4 through a series of salt bridges: Lys303 with Asp457, Asp342 with Lys464, Arg344 with Asp420, and Arg343 with Asp450 (Table 2 ▸). In molecule *B* all of these interactions are preserved, except for the Lys303–Asp457 and Asp97–Arg185 interactions (Table 2 ▸). In contrast, an interaction between residues Lys434 and Asp337 is observed (Table 2 ▸). In both molecules, no salt-bridge interactions were found between β-trefoil domains 1 and 4.

We extended our analysis of salt-bridge interactions to previously reported crystal structures of fascin1 in its free form [PDB entries 1dfc (Sedeh *et al.*, 2010 ▸), 3llp (Chen *et al.*, 2010 ▸) and 3p53 (Jansen *et al.*, 2011 ▸)]. All salt bridges across chains *A* and *B* in each crystal structure are collected in Supplementary Tables S4–S6. A comprehensive summary of the salt-bridge interactions observed across these structures is provided in Table 2 ▸. Our analysis revealed several differences when compared with our structure, particularly in the distribution of salt-bridge interactions. The unliganded structures exhibit the following numbers of salt bridges (Table 2 ▸): PDB entry 1dfc, 81 in chain *A*, 94 in chain *B*; PDB entry 3llp, 91 in chain *A*, 99 in chain *B*; PDB entry 3p53, 97 in chain *A*, 101 in chain *B*. While the overall number of salt bridges is comparable across all free fascin1 structures, the most significant differences lie in their distribution throughout the protein (Supplementary Tables S3–S6, Fig. 6 ▸ and Supplementary Fig. S5). Also, we observed marked differences in interdomain interactions (Table 2 ▸, Supplementary Fig. S5) so that only four interdomain salt bridges are conserved across all structures. Two of these bridges connect β-trefoil domains 3 and 4 (Arg343–Asp450 and Arg344–Asp420), while the remaining two link β-trefoil domain 2 to β-trefoil domain 1 (Arg224–Asp97) and β-trefoil domain 3 (Arg167–Asp290). Moreover, β-trefoil domain 3 engages in the highest number of salt-bridge interactions overall, suggesting its prominent role in stabilizing the protein structure. These findings highlight that despite the crystallization conditions being identical for all structures, differences in the spatial arrangement of salt bridges, especially interdomain salt bridges, could have implications for the functional dynamics of the protein.

### 3.4. Preliminary experiments on microcrystals of human fascin1

**Figure 7:**
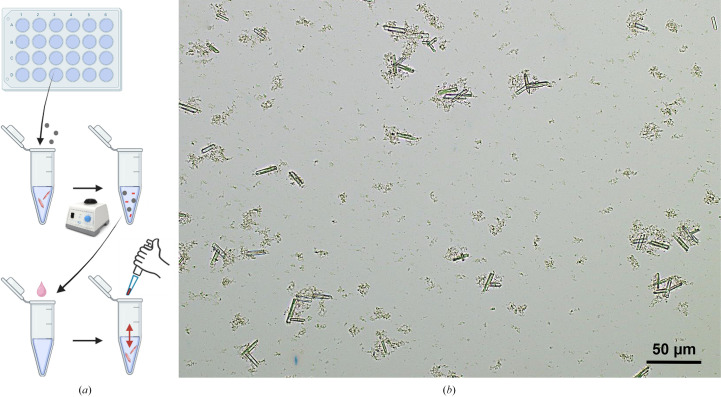
Microcrystals of human fascin1. (*a*) Schematic diagram of the microcrystallization process of the human fascin1 protein. (*b*) Microcrystals of human fascin1 were about 25–30 µm in size.

Our next step in human fascin1 research will be the study of its structural dynamics and allosteric properties. For this purpose, we will use time-resolved serial crystallography (TR-SX) experiments both at X-ray free-electron lasers (XFELs) and synchrotrons. Due to the nature of TR-SX experiments, crystal samples contain large amounts of microcrystals in the range of a few micrometres that are highly homogeneous in size. To this end, we optimized the human fascin1 crystallization conditions to obtain the required microcrystals. The best preliminary microcrystals were grown using the batch method with seeding (Fig. 7 ▸*a*) under optimization of the crystallization conditions in which large crystals were grown (see Section 2.6). They were needle-like microcrystals of about 25–30 µm in size (Fig. 7 ▸*b*) and with an ideal density for TR-SX experiments. These crystals were tested at a synchrotron and diffracted to ∼2.5 Å resolution, supporting their suitability for future time-resolved experiments.

## 4. Discussion

The structural flexibility of human fascin1 and its dynamic interaction with actin filaments allows it to cross-link actin, forming cellular protrusions such as filopodia and invadopodia which are key to cancer cell migration and metastasis (Jayo & Parsons, 2010 ▸; Hashimoto *et al.*, 2005 ▸). Structural studies, including crystallography and cryo-electron microscopy, reveal that fascin1 undergoes conformational changes upon actin binding, enhancing its bundling activity and rearranging its actin-binding sites (Gong *et al.*, 2025 ▸; Chung *et al.*, 2022 ▸). In this study, we present the X-ray structure of human fascin1 at a resolution of 2.2 Å. The high quality of the structure enabled us to model all protein residues continuously, making it the first structure in which both molecules in the asymmetric unit are fully modeled. Our structure and comparative analysis suggest the existence of various conformational substates prior to ligand binding and subsequent biochemical activity, supporting a mechanism of conformational selection that from an equilibrium standpoint may give rise to cooperative effects by revealing distinct substates with different functional properties, thereby providing high-resolution insights into structure–function relationships. From an equilibrium standpoint, the presence of these distinct conformational substates, which may display different functional properties, suggests that cooperative effects could arise from a conformational selection mechanism initiated by ligand binding.

Salt bridges are critical mediators of allosteric regulation, serving as dynamic interactions that stabilize specific protein conformations and facilitate communication between distant sites. By forming or breaking in response to ligand binding or other stimuli, salt bridges transmit structural and dynamic changes that shift the equilibrium between functional states. They often participate in networks that couple allosteric sites to active sites, modulate protein flexibility and fine-tune conformational transitions. For instance, in the penicillin-binding protein 2a responsible for β-lactam antibiotic resistance in *Staphylococcus aureus*, salt bridges play a key role in stabilizing the inactive ‘closed’ conformation of the active site (Otero *et al.*, 2013 ▸; Fishovitz *et al.*, 2014 ▸). Disruption of these interactions upon binding of allosteric effectors, such as β-lactam antibiotics or inhibitors such as ceftaroline, induces conformational changes that activate the enzyme or expose the active site (Otero *et al.*, 2013 ▸; Fishovitz *et al.*, 2014 ▸). Given the essential role of salt bridges in protein stability and conformational regulation, we hypothesized that similar interactions might influence the stability and conformational dynamics of fascin1. This variation in stability could be partially attributed to the formation or disruption of salt bridges within the protein. In this regard, we further explored the salt-bridge interactions within fascin1 to better understand their contribution to its stability and flexibility. Notably, we identified a range of salt bridges connecting the β-trefoil domains (Supplementary Tables S3–S6, Fig. 6 ▸ and Supplementary Fig. S5). This analysis revealed a notable absence of salt bridges between β-trefoil domains 1 and 4, which is in good agreement with the highly dynamic nature of this region revealed by structural studies (Gong *et al.*, 2025 ▸; Chung *et al.*, 2022 ▸) and molecular-dynamics simulations (Wu *et al.*, 2021 ▸). Based on these observations, it could be hypothesized that the spatial arrangement of β-trefoil domains 1 and 4 suggests that an interaction between these two domains could stabilize ABS1, the first actin-binding site. However, this stabilization might introduce rigidity into ABS1, potentially reducing its capacity to adjust during initial contact with the actin filament. Interestingly, all known inhibitors of fascin1 target this region, underlining its critical role in bundling activity. Stabilizing this flexible region would likely require substantial energy investment, as the protein would need to overcome its inherent flexibility to maintain conformations suitable for both actin binding and inhibitor interaction. Our analysis of salt-bridge patterns also suggests that these interactions play a key role in maintaining the structural integrity and functional flexibility of fascin1. Comparison of unliganded and inhibitor-bound structures highlights how inhibitors may affect these interactions (Supplementary Fig. S6), which could be crucial to their proposed antimetastatic effects. Further investigation into how these interdomain salt bridges modulate the actin-binding and inhibitor interactions of fascin1 could provide deeper insights into the structure–function relationship of the protein.

Like many proteins, human fascin1 employs allosteric regulation to modulate its activity through the binding of regulatory molecules, either activators or inhibitors, that alter the binding sites. However, it remains challenging to identify which specific regions of the protein structure are conformationally coupled. As a result, understanding of the molecular mechanisms underlying allosteric effects within human fascin1 has been constrained by the complexity of its interactions and the difficulty in obtaining structural and dynamic information on intermediate states using standard macromolecular crystallography. In this respect, time-resolved serial femtosecond X-ray crystallography (TR-SFX; Fromme *et al.*, 2020 ▸; Martin-Garcia *et al.*, 2016 ▸) offers a powerful tool to gain deeper insights into the function of fascin1 and its dynamic interaction with actin. All crystal structures reported for human fascin1 have been collected under cryogenic conditions (Sedeh *et al.*, 2010 ▸; Chen *et al.*, 2010 ▸; Jansen *et al.*, 2011 ▸). It is well established that both the cryoprotectant and the cooling process can induce conformational changes, reflecting only a limited subset of the possible conformations observed at room temperature. Experimental studies have shown these differences between room-temperature and cryogenic crystallo­graphic structures in several proteins (Liu *et al.*, 2013 ▸; Wolff *et al.*, 2023 ▸; Srinivas *et al.*, 2020 ▸; Suno *et al.*, 2018 ▸; Fenalti *et al.*, 2015 ▸; Ayan *et al.*, 2022 ▸; Pan *et al.*, 2022 ▸). The challenge of obtaining structural and dynamic information using standard macromolecular crystallography at cryogenic temperatures using synchrotrons has prevented researchers from observing this behavior from a structural standpoint. Therefore, by capturing molecular snapshots at different time points, TR-SFX can provide high-resolution structural information about the conformational changes that fascin1 undergoes during actin binding and inhibitor interaction. To this end, in addition to the complete high-resolution structure of free fascin1, here we present an initial attempt to identify crystallization conditions to tackle TR-SFX experiments with these proteins. We have managed to grow the first microcrystals of fascin1, and preliminary TR-SFX experiments are currently ongoing in our laboratories that could provide extremely relevant information about the conformational dynamics, function and regulation of fascin1, which would be of interest for the development of novel therapies.

## 5. Conclusions

This study presents the structure of human fascin1, fully modeling both molecules in the asymmetric unit and offering new insights into its conformational dynamics and structural plasticity. Our analysis highlights the critical role of salt bridges in stabilizing the fascin1 architecture while preserving the flexibility required for its actin-bundling activity. In particular, the flexible β-trefoil domains and their interdomain interactions are key to maintaining structural integrity and functional adaptability. The absence of salt bridges between β-trefoil domains 1 and 4 points to a dynamic region that could hinder stable ionic interactions, potentially impacting the stabilization and flexibility of ABS1 during initial contact with actin filaments.

Our study also suggests the presence of various conformational substates prior to ligand binding, supporting a mechanism of conformational selection. This indicates that cooperative effects may arise from this selection mechanism initiated by ligand binding. From the comprehensive analysis of salt-bridge interactions, we have identified connections between β-trefoil domains, with a notable absence of salt bridges between domains 1 and 4, suggesting that ionic interactions do play a relevant role in the regulation of the dynamics of fascin1.

The structural flexibility observed in our full-length fascin1 model highlights the need for time-resolved approaches to capture its dynamic behavior during actin and inhibitor binding. By revealing conformational heterogeneity in key functional regions, this work provides a foundation for future TR-SFX experiments aimed at uncovering the mechanistic basis of fascin1-mediated metastasis and informing the design of targeted inhibitors.

## Supplementary Material

PDB reference: human fascin1, 9fn6

Supplmentary Figures and Tables. DOI: 10.1107/S2053230X25005254/uf5017sup1.pdf

## Figures and Tables

**Table 1:** X-ray data-collection and refinement statistics Values in parentheses are for the outer shell.

Data-collection statistics	
X-ray source/beamline	ALBA/BL-13 XALOC
Wavelength (Å)	0.98
Temperature (K)	100
Detector	PILATUS X 6M
Space group	*I*121
*a*, *b*, *c* (Å)	114.566, 70.804, 122.210
α, β, γ (°)	90.00, 92.78, 90.00
Resolution range (Å)	43.42–2.20 (2.20–2.25)
No. of unique reflections	47005
Completeness (%)	99.16 (99.75)
CC_1/2_ (%)	99.5 (91.8)
Multiplicity	6.7 (6.8)
*R*_merge_ (%)	10.6 (54.1)
〈*I*/σ(*I*)〉	4.40
Overall *B* factor from Wilson plot (Å^2^)	48.24
Refinement statistics	
Resolution range (Å)	43.42–2.20 (2.20–2.25)
No. of reflections, working set	47005
No. of reflections, test set	3436
*R*_work_/*R*_free_ (%)	23.4/30.4
No. of non-H atoms	
Protein	7769
Water	308
Other	0
R.m.s. deviations	
Bond lengths (Å)	0.012
Bond angles (°)	1.823
Average *B* factors (Å^2^)	
All atoms	38.06
Protein	37.78
Water	45.26
Ramachandran plot	
Favored (%)	91
Allowed (%)	7
Outliers (%)	2
PDB code	9fn6

**Table 2:** Analysis of salt-bridge interactions in human fascin1

		This study		PDB entry 1dfc		PDB entry 3llp		PDB entry 3p53	
		Chain *A*	Chain *B*	Chain *A*	Chain *B*	Chain *A*	Chain *B*	Chain *A*	Chain *B*
No. of salt bridges	β-Trefoil domain 1	24	15	15	15	13	20	16	18
	β-Trefoil domain 2	25	36	27	38	35	33	32	28
	β-Trefoil domain 3	27	27	18	19	26	27	26	34
	β-Trefoil domain 4	27	23	21	22	17	19	23	21
Total No. of salt bridges		103	101	81	94	91	99	97	101
Salt bridges linking domains†	Glu8–Arg300	N	N	N	N	N	N	N	Y
	Lys47–Glu215	N	Y	N	N	N	N	N	N
	Asp97–Arg185	Y	N	Y	Y	N	N	N	N
	Asp97–Arg224	Y	Y	Y	Y	Y	Y	Y	Y
	Arg167–Asp290	Y	Y	Y	Y	Y	Y	Y	Y
	Lys303–Asp457	Y	N	N	N	Y	N	Y	Y
	Asp337–Lys434	N	Y	N	N	N	N	N	N
	Asp342–Lys464	Y	Y	Y	Y	Y	Y	Y	Y
	Arg343–Asp420	N	N	N	N	Y	Y	N	Y
	Arg343–Asp450	Y	Y	Y	Y	Y	Y	Y	Y
	Arg344–Asp420	Y	Y	Y	Y	Y	Y	Y	Y

† For clarity, all salt-bridge interactions are color-coded according to the corresponding β-trefoil domains in chain *A* of the fascin1 structures presented in this study: magenta for β-trefoil domain 1, orange for β-trefoil domain 2, green for β-trefoil domain 3 and blue for β-trefoil domain 4.
