# Supplementary material for: Structural insights into full-length human fascin1: a target for cancer treatment
Source: Acta Crystallogr F Struct Biol Commun. 2025 Jun 27;81(Pt 7):319–31. doi: 10.1107/S2053230X25005254 (PMC12210192; doi:10.1107/S2053230X25005254)
Supplement: Supplementary file 1 [file f-81-00319-sup1.pdf]

STRUCTURAL BIOLOGY
COMMUNICATIONS

Volume 81 (2025)

Supporting information for article:

Structural insights into full-length human fascin1: a target for cancer treatment

Lucía Giraldo-Ruiz, Isabel Quereda-Moraleda, Alice Grieco, Javier Ruiz-Sanz, Irene Luque and Jose Manuel Martin-Garcia

Supplementary Figures

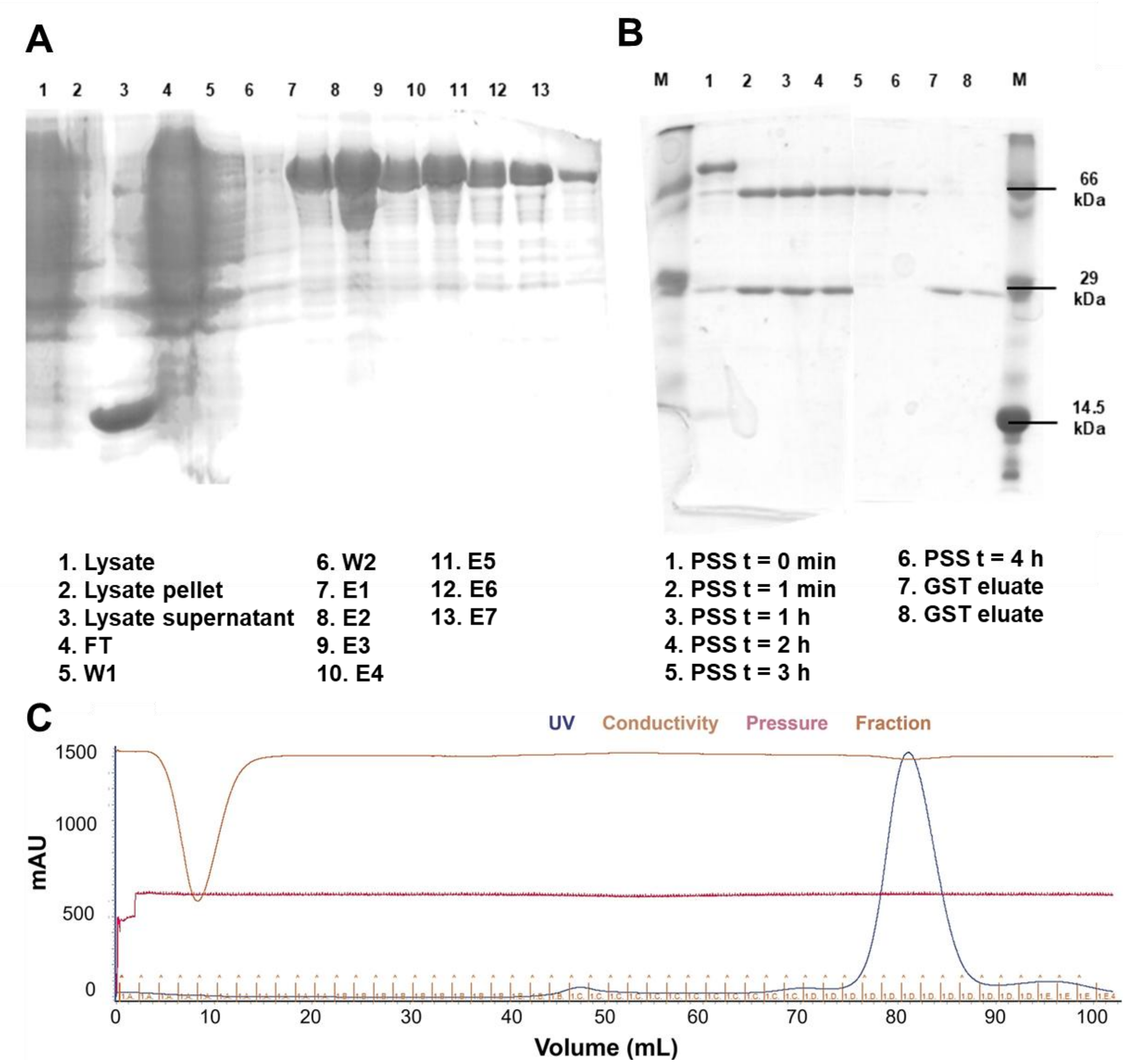

Figure S1: Expression and purification of the human Fascin 1 protein. A) SDS-PAGE gel showing the cell lysis and first GSH-affinity chromatography steps. Protein mostly eluted in fractions E1 to E7. **B)** SDS-PAGE gel showing the cleavage with the protease PSS and the second GSH-affinity chromatography steps. The bands were visualized by Coomassie stain. FT: flowthrough; W: wash; E: elution; M: protein marker; PSS: Pre-Scission Protease®. **C)** Size-exclusion chromatography performed using a HiLoad 16/600 Superdex 200 prep grade column in an ÄKTA go™ system.

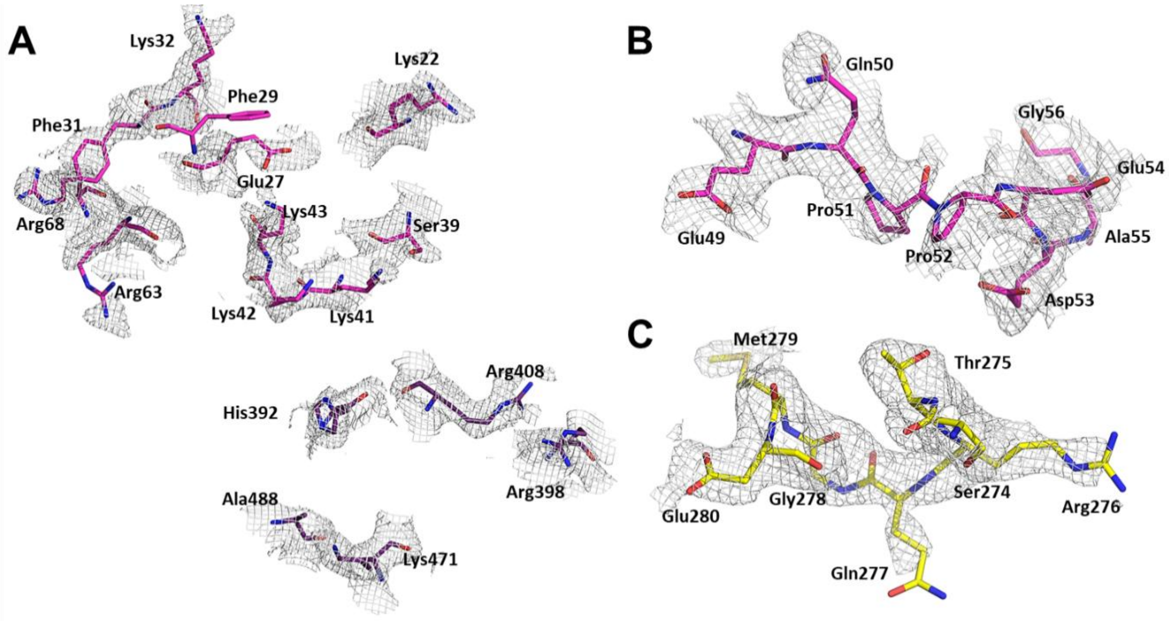

Figure S2: Electron density maps of Fascin1. A) $2mF_o$-DF_c electron density map contoured at 1 σ for the residues in ABS1 of chain B. **B)** $2mF_o$-DF_c electron density map contoured at 1 σ for residues 49-56 within β-trefoil domain 1. **C)** $2mF_o$-DF_c electron density map contoured at 1 σ for residues 274-280 within β-trefoil domain 3.

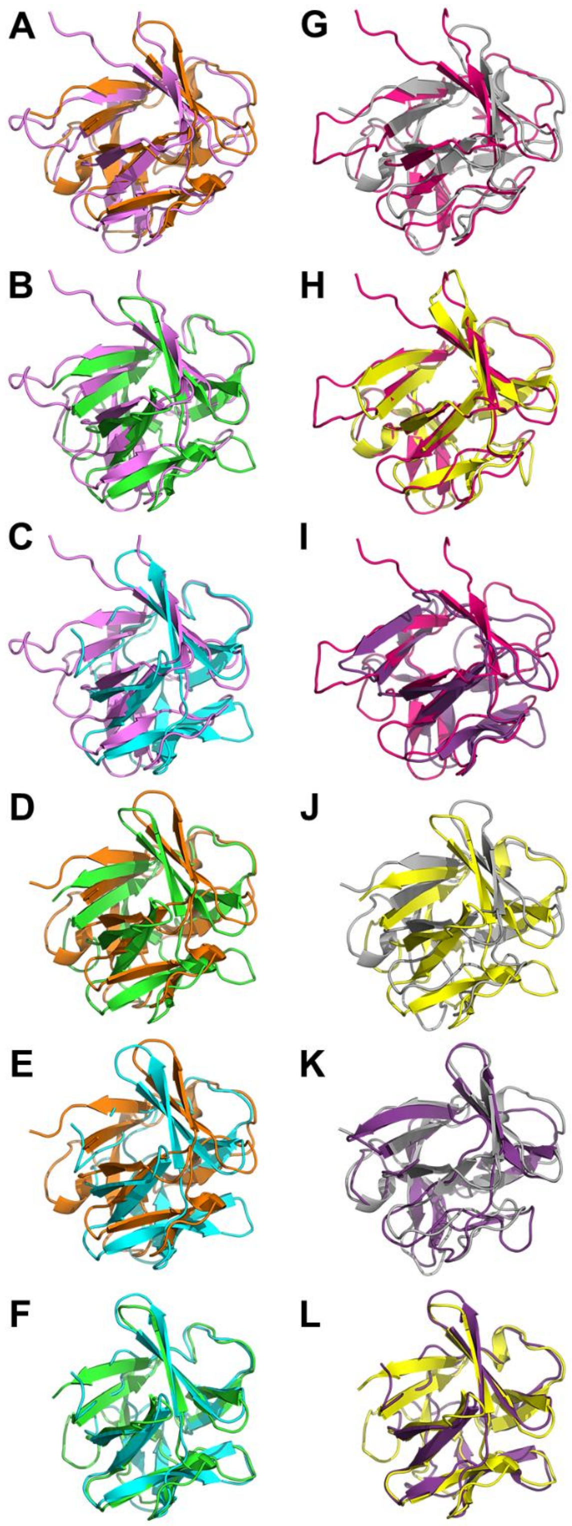

Figure S3: Pairwise structural comparisons of the β-trefoil domains in chains A and B. Panels A–F show comparisons among β-trefoil domains 1–4 of chain A: **A)** domain 1 vs domain 2, **B)** domain 1 vs domain 3, **C)** domain 1 vs domain 4, **D)** domain 2 vs domain 3, **E)** domain 2 vs domain 4, and **F)** domain 3 vs domain 4. Panels G–L display analogous comparisons for chain B: **G)** domain 1 vs domain 2, **H)** domain 1 vs domain 3, **I)** domain 1 vs domain 4, **J)** domain 2 vs domain 3, **K)** domain 2 vs domain 4, and **L)** domain 3 vs domain 4. Color codes are as follows: for chain A, domain 1 is magenta, domain 2 is orange, domain 3 is green, and domain 4 is cyan; for chain B, domain 1 is pink, domain 2 is gray, domain 3 is yellow, and domain 4 is purple.

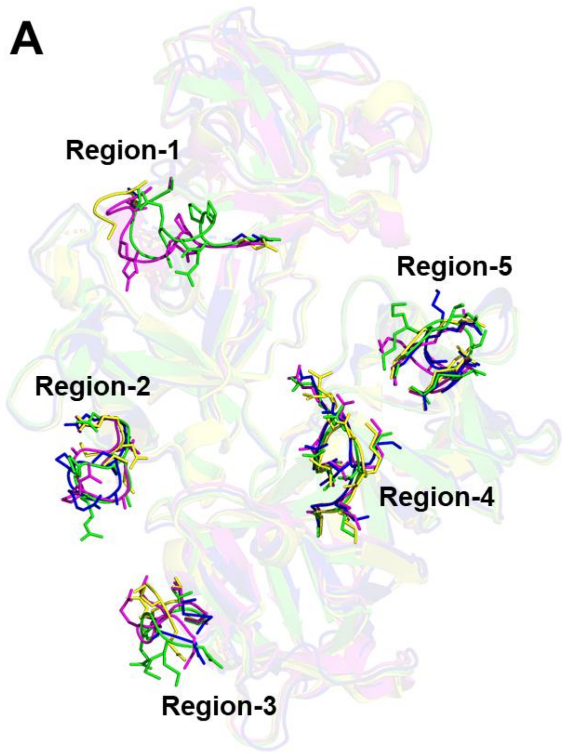

Figure S4: Structural superimposition of human Fascin1 structures. A) Superimposition of chain A from the structure presented in this study (yellow) with crystal structures PDB 3LLP (yellow), 1DFC (blue), and 3P53 (magenta). **B)** Superimposition of chain A from the structure presented in this study (orange) with crystal structures PDB 3LLP (purple), 1DFC (cyan), and 3P53 (grey). In both panels, regions exhibiting significant conformational changes are highlighted as sticks: region 1 (49–59), region 2 (156–162), region 3 (274–280), region 4 (298–305), and region 5 (397–404).

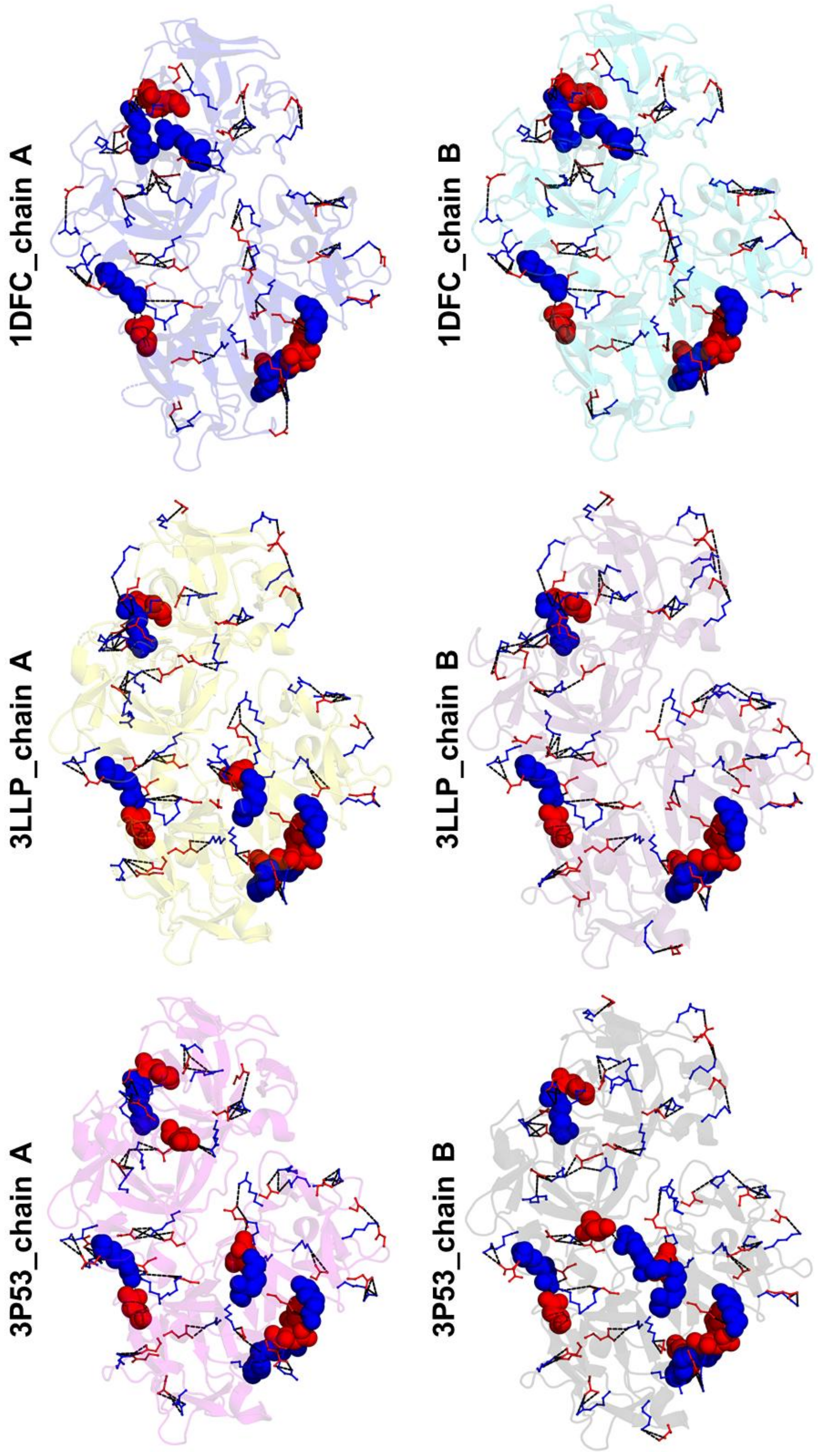

Figure S5: Salt bridge interactions in human Fascin1 protein. Balls and stick representation of all salt bridges found in molecules A (left panels) and B (right panels) in the ASU of the crystal structures previously reported (PDB 1DFC, 3LLP, and 3P53). Positive residues (Arg and Lys) are shown in blue and negative residues (Glu and Asp) are in red. Salt bridges are shown as black dashes. Residues involved in the interdomain interactions are shown as spheres. Fascin1 molecules are shown as cartoon using the same color code as in Figure S4.

Supplementary Tables

Table S1. R.M.S.D.s comparison in chains A and B of the Fascin1 structure reported in this study.

	**Chain A**	**Chain B**
β-trefoil domain 1 vs β-trefoil domain 2	3.441 Å	3.544 Å
β-trefoil domain 1 vs β-trefoil domain 3	0.673 Å	0.825 Å
β-trefoil domain 1 vs β-trefoil domain 4	2.706 Å	2.497 Å
β-trefoil domain 2 vs β-trefoil domain 3	9.605 Å	9.621 Å
β-trefoil domain 2 vs β-trefoil domain 4	2.122 Å	1.992 Å
β-trefoil domain 3 vs β-trefoil domain 4	0.801 Å	0.756 Å

Table S2. Salt bridge interactions in the Fascin1 protein structure reported in this study

Chain A			Chain B		
Residue / atom	Residue / atom	Distance (Å)	Residue / atom	Residue / atom	Distance (Å)
Lys22 / NZ	Glu116 / OE1	5.0	Lys22 / NZ	Glu116 / OE1	2.8
--	--	--	Lys22 / NZ	Glu116 / OE2	4.4
Lys43 / NZ	Glu27 / OE2	4.6	Lys43 / NZ	Glu27 / OE1	3.8
--	--	--	Lys43 / NZ	Glu27 / OE2	2.3
--	--	--	Lys47 / NZ	Glu215 / OE1	4.9
--	--	--	Lys47 / NZ	Glu215 / OE2	3.7
Arg63 / NH1	Glu49 / OE1	4.6	--	--	--
Arg63 / NH1	Glu49 / OE2	3.3	--	--	--
--	--	--	Arg68 / NH1	Glu81 / OE2	4.9
Arg68 / NH1	Glu83 / OE1	4.7	Arg68 / NH1	Glu83 / OE1	4.8
Arg68 / NH1	Glu83 / OE2	4.0			
Arg68 / NH2	Glu83 / OE1	4.6	Arg68 / NH2	Glu83 / OE1	4.8
Arg68 / NH2	Glu83 / OE2	4.6	--	--	--
Lys74 / NZ	Asp73 / OD1	4.1	--	--	--
Lys74 / NZ	Asp73 / OD2	4.1	--	--	--
Lys74 / NZ	Asp75 / OD1	4.1	--	--	--
Lys74 / NZ	Asp75 / OD2	2.8	--	--	--
Arg82 / NH2	Asp88 / OD2	4.5	Arg82 / NH1	Asp88 / OD2	4.8
--	--	--	Arg82 / NH2	Asp88 / OD1	4.6
--	--	--	Arg82 / NH2	Asp88 / OD2	3.3
Arg90 / NH1	Glu54 / OE2	4.2	Arg90 / NH2	Glu54 / OE1	4.3
--	--	--	Arg90 / NH2	Glu54 / OE1	
Arg90 / NH2	Glu54 / OE2	3.4	--	--	--
Arg90 / NH1	Glu106 / OE1	4.7	Arg90 / NH1	Glu106 / OE1	3.4
--	--	--	Arg90 / NH1	Glu106 / OE2	5.0
Arg90 / NH2	Glu106 / OE1	3.3	--	--	--
Arg90 / NH2	Glu106 / OE2	4.5	--	--	--
Arg100 / NH1	Asp98 / OD2	4.5	--	--	--
Arg100 / NH2	Asp98 / OD1	4.5	--	--	--
Arg100 / NH2	Asp98 / OD2	3.0	--	--	--
Arg118 / NH1	Asp117 / OD1	3.4	--	--	--
Lys131 / NZ	Asp98 / OD1	4.3	--	--	--
Lys131 / NZ	Asp98 / OD2	4.6	--	--	--
--	--	--	Arg151 / NH1	Asp168 / OD1[A]	4.0
--	--	--	Arg151 / NH1	Asp168 / OD2[A]	4.6
--	--	--	Arg151 / NH2	Asp168 / OD1	4.3

Arg167 / NH1	Asp174 / OD1	2.4	Arg167 / NH1	Asp174 / OD1	3.4
Arg167 / NH1	Asp174 / OD2	3.3	Arg167 / NH1	Asp174 / OD2	3.1
Arg167 / NH2	Asp174 / OD2	4.1	Arg167 / NH2	Asp174 / OD1	4.6
--	--	--	Arg167 / NH2	Asp174 / OD2	4.7
Arg167 / NH1	Asp290 / OD1	4.9	--	--	--
Arg167 / NH2	Asp290 / OD2	2.9	Arg167 / NH2	Asp290 / OD1	3.7
Arg167 / NH2	Asp290 / OD1	3.0	Arg167 / NH2	Asp290 / OD2	3.0
Arg185 / NH2	Asp97 / OD1	5.0	Arg185 / NH1[A]	Asp183 / OD1	4.6
Arg194 / NH1	Asp161 / OD1	2.8	Arg194 / NH1[B]	Asp161 / OD1	3.1
Arg194 / NH1	Asp161 / OD2	4.0	Arg194 / NH1[B]	Asp161 / OD2	2.7
Arg194 / NH2	Asp161 / OD1	3.6	Arg194 / NH2	Asp161 / OD1	2.6
--	--	--	Arg194 / NH2	Asp161 / OD2	3.8
--	--	--	Arg194 / NH1[B]	Asp192 / OD2	4.4
Arg194 / NH2	Asp192 / OD1	4.7	Arg194 / NH2	Asp192 / OD1	4.5
Arg194 / NH2	Asp192 / OD2	2.8	Arg194 / NH2	Asp192 / OD2	3.0
Arg197 / NH1	Asp192 / OD2	3.0	--	--	--
Arg197 / NH1	Asp199 / OD1	3.6	Arg197 / NH1[A]	Asp199 / OD1[B]	4.0
--	--	--	Arg197 / NH1[A]	Asp199 / OD2[B]	2.9
Arg197 / NH2	Glu207 / OE1	3.2	Arg197 / NH1[B]	Glu207 / OE1[A]	3.4
Arg197 / NH2	Glu207 / OE2	4.3	Arg197 / NH1[B]	Glu207 / OE1[A]	4.9
--	--	--	Arg197 / NH1[B]	Glu207 / OE2[A]	1.9
--	--	--	Arg197 / NH1[B]	Glu207 / OE2[A]	3.2
--	--	--	Arg197 / NH1[B]	Glu207 / OE2[B]	5.0
--	--	--	Arg197 / NH2[A]	Glu207 / OE1[B]	3.2
--	--	--	Arg197 / NH2[A]	Glu207 / OE1[B]	5.0
--	--	--	Arg197 / NH2[A]	Glu207 / OE2[B]	3.9
--	--	--	Arg197 / NH2[B]	Glu207 / OE2[A]	4.1
--	--	--	Arg197 / NH2[A]	Glu207 / OE2[A]	4.3
Arg217 / NH2	Glu215 / OE1	4.8	--	--	--
Arg224 / NH1	Asp97 / OD1	2.5	Arg224 / NH1	Asp97 / OD1[A]	2.4
Arg224 / NH1	Asp97 / OD2	4.0	Arg224 / NH1	Asp97 / OD1[B]	4.9
Arg224 / NH2	Asp97 / OD1	3.8	Arg224 / NH1	Asp97 / OD2[A]	4.3
Arg224 / NH2	Asp97 / OD2	4.5	Arg224 / NH2	Asp97 / OD1[A]	3.6
--	--	--	Arg224 / NH2	Asp97 / OD2[A]	4.6
Arg229 / NH1	Asp225 / OD2	4.4	--	--	--
Arg229 / NH2	Asp225 / OD1	3.8	--	--	--
Arg229 / NH2	Asp225 / OD2	2.7	--	--	--
Arg229 / NH2	Glu227 / OE1	4.8	--	--	--
--	--	--	Lys247 / NZ	Glu 215/OE1	4.9
--	--	--	Lys247 / NZ	Glu 215/OE2	3.7

Arg271 / NH1	Asp286 / OD1	4.7	Arg271 / NH1	Asp 286/OD1	4.2
Arg271 / NH2	Asp286 / OD1	3.3	Arg271 / NH1	Asp 286/OD2	4.6
Arg271 / NH2	Asp286 / OD2	3.4	Arg271 / NH2	Asp 286/OD1	2.8
--	--	--	Arg271 / NH2	Asp 286/OD2	2.8
Arg276 / NH1	Glu287 / OE1	3.2	Arg276 / NH1	Glu 287/OE1	3.2
Arg276 / NH1	Glu287 / OE2	4.8	Arg276 / NH1	Glu 292/OE1	3.5
Arg276 / NH1	Glu287 / OE2	4.9	Arg276 / NH1	Glu 292/OE2	2.9
Arg276 / NH2	Glu287 / OE1	2.4	Arg276 / NH2	Glu 287/OE1	2.7
Arg276 / NH2	Glu287 / OE2	4.2	Arg276 / NH2	Glu 287/OE2	4.2
Arg276 / NH2	Glu287 / OE2	4.5	--	--	--
Arg276 / NH2	Glu292 / OE1	3.0	Arg276 / NH2	Glu 292/OE1	4.6
Arg276 / NH2	Glu292 / OE2	3.2	Arg276 / NH2	Glu 292/OE2	4.5
Lys303 / NZ	Asp457 / OD1	4.7	--	--	--
Lys304 / NZ	Asp337 / OD1	3.0	Lys304 / NZ	Asp 337/OD1	3.0
Lys304 / NZ	Asp337 / OD2	4.0	Lys304 / NZ	Asp 337/OD2	4.8
Lys304 / NZ	Glu297 / OE1	2.6	--	--	--
Lys304 / NZ	Glu297 / OE2	4.8	--	--	--
--	--	--	Arg308 / NH1	Glu 297/OE2	4.9
--	--	--	Lys330 / NZ	Glu 297/OE1	2.6
--	--	--	Lys330 / NZ	Glu 297/OE2	4.8
Arg341 / NH2	Glu339 / OE2	4.3	Arg341 / NH2	Glu 339/OE2	5.0
Arg343 / NH1	Asp450 / OD2	4.9	Arg343 / NH1	Asp 450/OD2	4.9
Arg343 / NH2	Asp450 / OD1	4.8	Arg343 / NH2	Asp 450/OD1	4.9
Arg343 / NH2	Asp450 / OD2	2.7	Arg343 / NH2	Asp 450/OD2	2.8
Arg344 / NH1	Asp420 / OD1	3.2	Arg344 / NH1	Asp 420/OD1	3.0
Arg344 / NH1	Asp420 / OD2	4.0	Arg344 / NH1	Asp 420/OD2	3.6
Arg344 / NH2	Asp420 / OD1	3.7	Arg344 / NH2	Asp 420/OD1	3.8
Arg344 / NH2	Asp420 / OD2	4.0	Arg344 / NH2	Asp 420/OD2	4.9
Arg348 / NH1	Asp337 / OD2	5.0	Arg348 / NH1	Asp337 / OD2	4.6
Lys379 / NZ	Glu288 / OE1	3.2	Lys379 / NZ	Glu288 / OE1	3.4
Lys379 / NZ	Glu288 / OE2	2.8	Lys379 / NZ	Glu288 / OE2	2.7
Arg389 / NH1	Glu492 / OE1	5.0	Arg389 / NH1	Glu492 / OE1	3.7
Arg389 / NH1	Glu492 / OE2	3.8	Arg389 / NH1	Glu492 / OE2	3.6
Arg398 / NH1	Asp405 / OD1	2.5	Arg398 / NH1	Asp405 / OD1	2.5
Arg398 / NH1	Asp405 / OD2	3.4	Arg398 / NH1	Asp405 / OD2	3.4
Arg398 / NH2	Asp405 / OD1	3.2	Arg398 / NH2	Asp405 / OD1	3.6
Arg398 / NH2	Asp405 / OD2	4.2	Arg398 / NH2	Asp405 / OD2	4.2
Lys399 / NZ	Asp427 / OD2	3.8	--	--	--
Arg408 / NH1[A]	Asp405 / OD2	4.9	--	--	--
Arg408 / NH2[A]	Asp405 / OD2	2.7	--	--	--

Arg408 / NH1[B]	Asp412 / OD1	4.0	Arg408 / NH1	Asp412 / OD1	4.6
Arg408 / NH1[B]	Asp412 / OD2	2.5	Arg408 / NH1	Asp412 / OD2	4.9
Arg408 / NH2[B]	Asp412 / OD1	3.1	--	--	--
Arg408 / NH2[B]	Asp412 / OD2	3.3	--	--	--
Lys426 / NZ	Glu417 / OE1	2.8	Lys426 / NZ	Glu417 / OE1	3.3
Lys426 / NZ	Glu417 / OE2	4.4	Lys426 / NZ	Glu417 / OE2	4.0
Lys431 / NZ	Asp446 / OD1	4.9	Lys431 / NZ	Asp446 / OD1	2.9
Lys431 / NZ	Asp446 / OD2	3.4	--	--	--
--	--	--	Arg434 / NH1	Asp337 / OD2	4.6
Lys460 / NZ	Asp457 / OD2	4.1	Lys460 / NZ	Asp457 / OD1	4.7
--	--	--	Lys460 / NZ	Asp457 / OD2	2.7
Lys464 / NZ	Asp342 / OD2	4.1	Lys464 / NZ	Asp342 / OD1	2.9
--	--	--	Lys464 / NZ	Asp342 / OD2	4.4
Lys464 / NZ	Glu454 / OE1	4.2	Lys464 / NZ	Glu454 / OE1	3.4
Arg468 / NH1	Glu483 / OE2	3.4	--	--	--
Arg468 / NH2	Glu483 / OE1	4.9	Arg468 / NH2	Glu483 / OE1	4.4
Arg468 / NH2	Glu483 / OE2	2.9	--	--	--
Lys471 / NZ	Asp486 / OD1	4.8	Lys471 / NZ	Asp486 / OD1	4.8
Lys471 / NZ	Asp486 / OD2	4.0	Lys471 / NZ	Asp486 / OD2	4.1
Lys479 / NZ	Asp438 / OD2	4.0	Lys479 / NZ	Asp438 / OD1	4.4
--	--	--	Lys479 / NZ	Asp438 / OD2	3.6
Lys479 / NZ	Asp473 / OD2	4.8	Lys479 / NZ	Asp473 / OD2	4.4

For clarity, all salt bridge interactions observed in both chains A and B are color-coded according to the corresponding β-trefoil domains in chain A of the Fascin1 structure presented in this study: magenta for β-trefoil domain 1, orange for β-trefoil domain 2, green for β-trefoil domain 3, and cyan for β-trefoil domain 4.

Table S3. Salt bridge interactions in the Fascin 1 protein structure PDB 1DFC

Chain A			Chain B		
**Residue / atom**	**Residue / atom**	**Distance (Å)**	**Residue / atom**	**Residue / atom**	**Distance (Å)**
Lys43 / NZ	Glu27 / OE1	4.8	Lys43 / NZ	Glu27 / OE1	4.9
Lys43 / NZ	Glu27 / OE2	3.8	Lys43 / NZ	Glu27 / OE2	4.0
Arg63 / NH1	Glu49 / OE1	4.5	Arg63 / NH1	Glu49 / OE1	4.5
Arg63 / NH1	Glu49 / OE2	2.8	Arg63 / NH1	Glu49 / OE2	2.8
Arg63 / NH2	Glu49 / OE2	4.7	Arg63 / NH2	Glu49 / OE2	4.6
Arg68 / NH1	Glu81 / OE2	4.5	Arg68 / NH1	Glu81 / OE2	4.5
Arg68 / NH1	Glu83 / OE1	4.8	Arg68 / NH1	Glu83 / OE1	4.6
Arg68 / NH1	Glu83 / OE2	4.4	Arg68 / NH1	Glu83 / OE2	4.3
Arg68 / NH2	Glu83 / OE1	4.5	Arg68 / NH2	Glu83 / OE1	4.3
Arg68 / NH2	Glu83 / OE2	4.8	Arg68 / NH2	Glu83 / OE2	4.7
Arg82 / NH1	Asp108 / OD1	4.4	Arg82 / NH1	Asp108 / OD1	4.4
Arg90 / NH1	Glu106 / OE1	2.7	Arg90 / NH1	Glu106 / OE1	2.6
Arg90 / NH1	Glu106 / OE2	2.9	Arg90 / NH1	Glu106 / OE2	2.8
Arg90 / NH2	Glu106 / OE1	4.1	Arg90 / NH2	Glu106 / OE1	4.0
Arg90/ NH2	Glu106 / OE2	4.4	Arg90/ NH2	Glu106 / OE2	4.3
Arg149 / NH1	Asp251 / OD2	4.7	Arg149 / NH1	Asp251 / OD2	4.7
Lys150 / NZ	Asp168 / OD1	2.5	Lys150 / NZ	Asp168 / OD1	2.6
Lys150 / NZ	Asp168 / OD1	4.4	Lys150 / NZ	Asp168 / OD2	4.4
Arg151 / NH1	Asp168 / OD1	4.7	Arg151 / NH1	Asp168 / OD1	4.6
Arg151 / NH1	Asp168 / OD2	3.1	Arg151 / NH1	Asp168 / OD2	2.9
Arg151 / NH2	Asp168 / OD2	4.5	Arg151 / NH2	Asp168 / OD2	4.5
Arg167 / NH1	Asp174 / OD1	4.4	Arg167 / NH1	Asp174 / OD1	4.4
Arg167 / NH1	Asp174 / OD2	2.9	Arg167 / NH1	Asp174 / OD2	2.9
--	--	--	Arg167 / NH2	Asp174 / OD2	4.3
Arg167 / NH2	Asp290 / OD1	3.9	Arg167 / NH2	Asp290 / OD1	3.8
Arg167 / NH2	Asp290 / OD2	3.6	Arg167 / NH2	Asp290 / OD2	3.4
Arg185 / NH2	Asp98 / OD2	3.8	Arg185 / NH2	Asp98 / OD1	4.8
--	--	--	Arg185 / NH2	Asp98 / OD2	3.1
Arg194 / NH1	Asp161 / OD1	3.0	Arg194 / NH1	Asp161 / OD1	3.1
Arg194 / NH1	Asp161 / OD2	3.5	Arg194 / NH1	Asp161 / OD2	3.6
--	--	--	Arg194 / NH2	Asp161 / OD1	3.8
Arg194 / NH1	Asp192 / OD2	4.4	Arg194 / NH1	Asp192 / OD2	4.4
Arg194 / NH2	Asp192 / OD1	4.2	Arg194 / NH2	Asp192 / OD1	4.3
Arg194 / NH2	Asp192 / OD2	2.6	Arg194 / NH2	Asp192 / OD2	2.7
Arg197 / NH1	Asp199 / OD1	3.8	Arg197 / NH1	Asp199 / OD1	3.8
Arg197 / NH1	Asp199 / OD2	2.6	Arg197 / NH1	Asp199 / OD2	2.6

Arg197 / NH2	Asp199 / OD2	4.8	Arg197 / NH2	Asp199 / OD2	4.8
Arg197 / NH1	Glu207 / OE1	4.6	Arg197 / NH1	Glu207 / OE1	4.8
Arg197 / NH2	Glu207 / OE1	2.9	Arg197 / NH2	Glu207 / OE1	3.0
Arg197 / NH2	Glu207 / OE2	4.5	Arg197 / NH2	Glu207 / OE2	4.6
--	--	--	Arg201 / NH1	Asp 199/OD1	4.2
--	--	--	Arg205 / NH1	Glu207 / OE1	2.4
--	--	--	Arg205 / NH1	Glu207 / OE2	4.4
--	--	--	Arg205 / NH2	Glu207 / OE1	2.2
--	--	--	Arg205 / NH2	Glu207 / OE2	3.9
Arg224 / NH1	Asp97 / OD2	4.7	Arg224 / NH1	Asp97 / OD2	3.8
--	--	--	Arg224 / NH2	Asp97 / OD2	4.8
Arg229 / NH1	Asp225 / OD2	3.8	Arg229 / NH1	Asp225 / OD2	4.0
Arg229 / NH2	Asp225 / OD1	3.6	Arg229 / NH2	Asp225 / OD1	3.7
Arg229 / NH2	Asp225 / OD2	2.4	Arg229 / NH2	Asp225 / OD2	2.6
Arg229 / NH2	Glu227 / OE1	4.6	Arg229 / NH2	Glu227 / OE1	4.5
--	--	--	Arg271 / NH1	Asp286 / OD2	5.0
--	--	--	Arg271 / NH2	Asp286 / OD1	4.8
Arg271 / NH2	Asp286 / OD2	3.3	Arg271 / NH2	Asp286 / OD2	2.9
Lys304 / NZ	Asp337 / OD1	3.5	Lys304 / NZ	Asp337 / OD1	3.4
Lys304 / NZ	Asp337 / OD2	2.9	Lys304 / NZ	Asp337 / OD2	2.9
Arg308 / NH1	Glu297 / OE2	4.7	Arg308 / NH1	Glu297 / OE2	4.5
Lys330 / NZ	Glu297 / OE1	2.3	Lys330 / NZ	Glu297 / OE1	2.4
Lys330 / NZ	Glu297 / OE2	4.5	Lys330 / NZ	Glu297 / OE2	4.5
Arg341 / NH1	Glu339 / OE2	4.7	Arg341 / NH1	Glu339 / OE2	4.7
Arg341 / NH2	Glu339 / OE1	4.5	Arg341 / NH2	Glu339 / OE1	4.7
Arg341 / NH2	Glu339 / OE2	3.4	Arg341 / NH2	Glu339 / OE2	3.4
Arg341 / NH1	Asp372 / OD1	5.0	--	--	--
Arg343 / NH1	Asp 450/OD1	4.6	Arg343 / NH1	Asp 450/OD2	4.7
Arg343 / NH2	Asp 450/OD2	2.5	Arg343 / NH2	Asp 450/OD1	4.6
--	--	--	Arg343 / NH2	Asp 450/OD2	2.5
Arg344 / NH1	Asp420 / OD1	3.0	Arg344 / NH1	Asp420 / OD1	3.2
Arg344 / NH1	Asp420 / OD2	4.2	Arg344 / NH1	Asp420 / OD2	4.4
Arg344 / NH2	Asp420 / OD1	3.1	Arg344 / NH2	Asp420 / OD1	3.3
Arg344 / NH2	Asp420 / OD2	3.4	--	--	--
Lys379 / NZ	Glu288 / OE1	3.6	Lys379 / NZ	Glu288 / OE1	3.6
Lys379 / NZ	Glu288 / OE2	2.8	Lys379 / NZ	Glu288 / OE2	2.9
Arg389 / NH1	Glu492 / OE1	2.8	Arg389 / NH1	Glu492 / OE1	3.2
Arg389 / NH1	Glu492 / OE2	3.2	Arg389 / NH1	Glu492 / OE2	3.3
Arg389 / NH2	Glu492 / OE1	4.9	--	--	--
Arg398 / NH1	Asp405 / OD1	4.0	Arg398 / NH1	Asp405 / OD1	2.4

Arg398 / NH1	Asp405 / OD2	4.3	Arg398 / NH1	Asp405 / OD2	3.7
Arg398 / NH2	Asp405 / OD1	2.8	Arg398 / NH2	Asp405 / OD1	4.2
Arg398 / NH2	Asp405 / OD2	3.6	Arg398 / NH2	Asp405 / OD2	5.0
Arg408 / NH1	Asp405 / OD2	3.4	Arg408 / NH1	Asp405 / OD2	3.3
Arg408 / NH2	Asp405 / OD1	4.9	Arg408 / NH2	Asp405 / OD1	4.7
Arg408 / NH2	Asp405 / OD2	3.0	Arg408 / NH2	Asp405 / OD2	2.9
Lys426 / NZ	Glu417 / OE1	3.4	Lys426 / NZ	Glu417 / OE1	3.4
Lys426 / NZ	Glu417 / OE2	2.9	Lys426 / NZ	Glu417 / OE2	2.9
Lys460 / NZ	Asp457 / OD1	4.4	Lys460 / NZ	Asp457 / OD1	4.6
Lys460 / NZ	Asp457 / OD2	4.9	Lys460 / NZ	Asp457 / OD2	5.0
--	--	--	Lys460 / NZ	Glu492 / OE2	4.9
Lys464 / NZ	Glu454 / OE1	3.1	Lys464 / NZ	Glu454 / OE1	3.0
Lys464 / NZ	Glu454 / OE2	5.0	Lys464 / NZ	Glu454 / OE2	4.9
Lys464 / NZ	Asp342 / OD1	3.6	Lys464 / NZ	Asp342 / OD1	3.9
Arg468 / NH2	Glu483 / OE2	3.4	Arg468 / NH2	Glu483 / OE2	3.4
Lys471 / NZ	Asp486 / OD1	3.4	Lys471 / NZ	Asp486 / OD1	3.9
Lys471 / NZ	Asp486 / OD2	4.3	Lys471 / NZ	Asp486 / OD2	3.2
Lys479 / NZ	Asp438 / OD2	4.4	Lys479 / NZ	Asp438 / OD2	4.6
--	--	--	Lys479 / NZ	Asp473 / OD2	5.0

For clarity, all salt bridge interactions observed in both chains A and B of PDB 1DFC are color-coded according to the corresponding β-trefoil domains in chain A of the Fascin1 structures presented in this study: magenta for β-trefoil domain 1, orange for β-trefoil domain 2, green for β-trefoil domain 3, and cyan for β-trefoil domain 4.

Table S4. Salt bridge interactions in the Fascin1 structure PDB 3LLP

Chain A			Chain B		
Residue / atom	Residue / atom	Distance (Å)	Residue / atom	Residue / atom	Distance (Å)
Lys22 / NZ	Glu116 / OE1	5.0	Lys22 / NZ	Glu116 / OE2	4.4
--	--	--	Lys32 / NZ	Glu27 / OE2	4.2
--	--	--	Lys32 / NZ	Asp117 / OD2	4.6
Lys43 / NZ	Glu27 / OE1	2.6	Lys43 / NZ	Glu27 / OE1	2.6
--	Glu27 / OE2	3.6	Lys43 / NZ	Glu27 / OE2	3.7
Arg63 / NH1	Glu49 / OE2	4.4	--	--	--
Arg63 / NH2	Glu49 / OE2	2.9	--	--	--
--	--	--	Arg68 / NH1	Glu83 / OE1	4.8
Arg68 / NH1	Glu83 / OE2	4.1	Arg68 / NH1	Glu83 / OE2	4.6
Arg68 / NH2	Glu83 / OE2	3.7	Arg68 / NH2	Glu83 / OE2	4.3
Lys74 / NZ	Asp75 / OD2	4.7	Lys74 / NZ	Asp75 / OD2	4.4
--	--	--	Arg90 / NH1	Asp53 / OD1	3.3
--	--	--	Arg90 / NH1	Asp53 / OD2	3.4
Arg90 / NH1	Glu106 / OE1	3.8	Arg90 / NH1	Glu106 / OE1	4.8
Arg90 / NH1	Glu106 / OE2	4.9	--	--	--
--	--	--	Arg90 / NH2	Glu106 / OE1	3.4
--	--		Arg90 / NH2	Glu106 / OE2	4.1
--	--	--	Arg100 / NH1	Asp98 / OD1	4.8
--	--	--	Arg100 / NH1	Asp98 / OD2	4.6
Arg100 / NH2	Asp98 / OD1	4.4	--	--	--
Arg100 / NH2	Asp98 / OD2	3.3	--	--	--
--	--	--	Arg109 / NH1	Glu106 / OE2	4.7
Arg109 / NH2	Glu106 / OE2	5.0	--	--	--
Arg118 / NH1	Asp117 / OD1	3.6	Arg118 / NH1	Asp117 / OD1	3.8
--	--	--	Lys131 / NZ	Asp98 / OD1	3.8
--	--	--	Lys131 / NZ	Asp98 / OD2	2.8
Arg151 / NH1	Asp168 / OD1	3.8	Arg151 / NH1	Asp168 / OD1	4.5
Arg151 / NH2	Asp168 / OD1	4.6	Arg151 / NH2	Asp168 / OD1	4.2
Arg167 / NH1	Asp174 / OD2	4.8	--	--	--
Arg167 / NH2	Asp174 / OD1	3.0	--	--	--
Arg167 / NH2	Asp290 / OD2	3.4	--	--	--
Arg167 / NH1	Asp290 / OD1	3.8	Arg167 / NH1	Asp290 / OD1	3.0
Arg167 / NH1	Asp290 / OD2	2.8	Arg167 / NH1	Asp290 / OD2	3.3
Arg167 / NH2	Asp290 / OD1	3.1	Arg167 / NH2	Asp290 / OD1	3.8
Arg167 / NH2	Asp290 / OD2	3.4	Arg167 / NH2	Asp290 / OD2	2.8
--	--	--	Arg194 / NH1	Asp161 / OD1	2.9
Arg194 / NH1	Asp161 / OD2	3.1	--	--	--

Arg194 / NH2	Asp161 / OD1	4.3	Arg194 / NH2	Asp161 / OD1	3.1
Arg194 / NH2	Asp161 / OD2	2.6	--	--	--
Arg194 / NH1	Asp192 / OD2	4.8	Arg194 / NH1	Asp192 / OD2	4.5
Arg194 / NH2	Asp192 / OD1	4.8	Arg194 / NH2	Asp192 / OD1	4.6
Arg194 / NH2	Asp192 / OD2	3.1	Arg194 / NH2	Asp192 / OD2	3.0
Arg197 / NH1	Asp199 / OD2	4.9	Arg197 / NH1	Asp199 / OD2	4.9
Arg197 /NH2	Asp199 / OD1	4.7	Arg197 / NH2	Asp199 / OD1	3.7
Arg197 / NH2	Asp199 / OD2	3.4	Arg197 / NH2	Asp199 / OD2	2.9
Arg197 / NH1	Glu207 / OE1	2.8	--	--	--
Arg197 / NH1	Glu207 / OE2	3.6	Arg197 / NH1	Glu207 / OE2	4.3
--	--	--	Arg201 / NH1	Asp161 / OD1	4.3
--	--	--	Arg201 / NH1	Asp161 / OD2	4.2
--	--	--	Arg201 / NH2	Asp161 / OD2	4.3
Arg201 / NH1[A]	Asp199 / OD2	4.6	--	--	--
Arg201 / NH2[A]	Asp199 / OD1	4.0	--	--	--
Arg201 / NH2[A]	Asp199 / OD2	2.7	--	--	--
Arg217 / NH1	Glu215 / OE2	4.7	Arg217 / NH1	Glu215 / OE2	4.7
Arg217 / NH2	Glu215 / OE2	2.9	Arg217 / NH2	Glu215 / OE2	3.1
--	--	--	Arg217 / NH1	Glu252 / OE2	3.0
--	--	--	Arg217 / NH2	Glu252 / OE1	4.9
--	--	--	Arg217 / NH2	Glu252/ OE2	2.9
Arg224 / NH1	Asp97 / OD1	4.6	Arg224 / NH1	Asp97 / OD1	3.0
--	--	--	Arg224 / NH1	Asp97 / OD2	4.4
Arg224 / NH2	Asp97 / OD1	2.9	Arg224 / NH2	Asp97 / OD1	4.7
Arg224 / NH2	Asp97 / OD2	4.3	--	--	--
Arg224 / NH1	Glu215 / OE1	4.9	--	--	--
Arg229 / NH1	Asp225 / OD2	4.4	Arg229 / NH1	Asp225 / OD2	4.4
Arg229 / NH2	Asp225 / OD1	4.2	Arg229 / NH2	Asp225 / OD1	4.3
Arg229 / NH2	Asp225 / OD2	2.8	Arg229 / NH2	Asp225 / OD2	2.9
Arg229 / NH1	Glu227 / OE1	3.8	--	--	--
Arg229 / NH2	Glu227 / OE1	3.8	--	--	--
Arg229 / NH2	Glu227 / OE2	3.8	--	--	--
--	--	--	Lys247 / NZ	Asp251/OD1	2.9
--	--	--	Lys247 / NZ	Asp251/OD2	4.9
--	--	--	Lys224 / NZ	Glu252 / OE1	2.7
--	--	--	Lys224 / NZ	Glu252 / OE2	4.0
Arg276 / NH1	Glu287 / OE1	4.9	Arg276 / NH1	Glu287 / OE1	3.4
--	--	--	Arg276 / NH1	Glu287 / OE2	4.7
--	--	--	Arg276 / NH2	Glu287 / OE1	2.7
--	--	--	Arg276 / NH2	Glu287 / OE2	4.6

Arg276 / NH1	Glu292 / OE1	4.9	Arg276 / NH1	Glu292 / OE2	3.0
Arg276 / NH1	Glu292 / OE2	4.8	Arg276 / NH1	Glu292 / OE2	4.9
Arg300 / NH2[A]	Asp301 / OD1	4.6	--	--	--
Arg300 / NH2[A]	Asp301 / OD2	4.4	--	--	--
Lys303 / NZ	Asp457 / OD1	4.9	--	--	--
Lys303 / NZ	Asp457 / OD2	2.9	--	--	--
Lys304 / NZ	Asp337 / OD1	3.1	Lys304 / NZ	Asp337 / OD1	2.7
Lys304 / NZ	Asp337 / OD2	3.2	Lys304 / NZ	Asp337 / OD2	3.2
Arg308 / NH1	Glu297 / OE1	4.8	--	--	--
Arg308 / NH1	Glu297 / OE2	4.6	Arg308 / NH1	Glu297 / OE2	4.9
Lys330 / NZ	Glu297 / OE1	2.9	Lys330 / NZ	Glu297 / OE1	3.1
Lys330 / NZ	Glu297 / OE2	4.9	--	--	--
--	--	--	Lys330 / NZ	Asp299 / OD1	4.3
--	--	--	Lys330 / NZ	Asp299 / OD2	4.5
Arg341 / NH1	Glu339 / OE1	4.4	Arg341 / NH1	Glu339 / OE1	2.6
--	--	--	Arg341 / NH1	Glu339 / OE2	3.4
Arg341 / NH2	Glu339 / OE1	2.5	Arg341 / NH2	Glu339 / OE1	4.7
Arg341 / NH2	Glu339 / OE2	4.0	--	--	--
Arg343 / NH1	Asp420 / OD2	4.2	Arg343 / NH1	Asp420 / OD2	4.6
Arg343 / NH1	Asp450 / OD2	3.4	Arg343 / NH1	Asp450 / OD2	3.9
Arg343 / NH2	Asp450 / OD1	4.8	Arg343 / NH2	Asp450 / OD1	4.6
Arg343 / NH2	Asp450 / OD2	2.7	Arg343 / NH2	Asp450 / OD2	2.7
Arg344 / NH1	Asp420 / OD1	3.0	Arg344 / NH1	Asp420 / OD1	3.1
Arg344 / NH1	Asp420 / OD2	4.3	Arg344 / NH1	Asp420 / OD2	4.5
Arg344 / NH2	Asp420 / OD1	2.9	Arg344 / NH2	Asp420 / OD1	2.8
Arg344 / NH2	Asp420 / OD2	4.2	Arg344 / NH2	Asp420 / OD2	4.2
--	--	--	Lys353 / NZ	Glu368 / OE1	2.7
--	--	--	Lys353 / NZ	Glu368 / OE2	4.9
Lys379 / NZ	Glu288 / OE1	3.4	Lys379 / NZ	Glu 288 / OE1	3.9
Lys379 / NZ	Glu288 / OE2	2.7	Lys379 / NZ	Glu 288 / OE2	3.5
Arg389 / NH1	Glu492 / OE1	3.2	Arg389 / NH1	Glu492 / OE1	4.2
Arg389 / NH1	Glu492 / OE2	4.4	--	--	--
Arg398 / NH1	Asp405 / OD1	3.8	Arg398 / NH1	Asp405 / OD1	2.8
Arg398 / NH1	Asp405 / OD2	4.7	Arg398 / NH1	Asp405 / OD2	3.6
Arg398 / NH2	Asp405 / OD1	2.4	Arg398 / NH2	Asp405 / OD1	4.8
Arg398 / NH2	Asp405 / OD2	3.7	Arg398 / NH2	Asp405 / OD2	4.9
--	--	--	Lys399 / NZ	Asp427 / OD1	2.9
--	--	--	Lys399 / NZ	Asp427 / OD2	2.8
Arg408 / NH1	Asp405 / OD2	3.0	Arg408 / NH1	Asp405 / OD2	4.8
--	--	--	Arg408 / NH1	Asp412 / OD1	5.0

Lys426 / NZ	Glu417 / OE1	4.3	Lys426 / NZ	Glu417 / OE1	4.1
Lys426 / NZ	Glu417 / OE2	2.6	Lys426 / NZ	Glu417 / OE2	2.5
Lys431 / NZ	Asp446 / OD1	3.1	Lys431 / NZ	Asp446 / OD1	2.9
Lys431 / NZ	Asp446 / OD2	5.0	--	--	--
Lys460 / NZ	Glu492 / OE2	4.7	--	--	--
Lys464 / NZ	Asp342 / OD1	2.9	Lys464 / NZ	Asp342 / OD1	3.1
Lys464 / NZ	Asp342 / OD2	4.4	Lys464 / NZ	Asp342 / OD2	4.5
Lys464 / NZ	Glu454 / OE2	4.3	Lys464 / NZ	Glu454 / OE2	3.9
--	--	--	Arg468 / NH1	Glu483 / OE2	4.5
Arg468 / NH2	Glu483 / OE1	4.6	--	--	--
--	--	--	Arg468 / NH2	Glu483 / OE2	3.1
--	--	--	Lys471 / NZ	Asp486 / OD1	4.8
Lys479 / NZ	Asp473 / OD2	2.9	Lys479 / NZ	Asp473 / OD2	3.6

For clarity, all salt bridge interactions observed in both chains A and B of PDB 3LLP are color-coded according to the corresponding β-trefoil domains in chain A of the Fascin1 structures presented in this study: magenta for β-trefoil domain 1, orange for β-trefoil domain 2, green for β-trefoil domain 3, and cyan for β-trefoil domain 4.

Table S5. Salt bridge interactions in the Fascin1 structure PDB 3P53

Chain A			Chain B		
**Residue / atom**	**Residue / atom**	**Distance (Å)**	**Residue / atom**	**Residue / atom**	**Distance (Å)**
--	--	--	Lys22 / NZ	Glu116 / OE1	3.3
--	--	--	Lys22 / NZ	Glu116 / OE2	4.4
--	--	--	Lys43 / NZ	Glu27 / OE2	4.9
Arg63 / NH1[A]	Glu49 / OE2	4.3	Arg63 / NH1	Glu49 / OE2	4.8
Arg63 / NH1[B]	Glu49 / OE2	4.9	--	--	--
Arg63 / NH2[B]	Glu49 / OE1	4.7	Arg63 / NH2	Glu49 / OE1	4.6
Arg63 / NH2[B]	Glu49 / OE2	2.6	Arg63 / NH2	Glu49 / OE2	3.0
Arg68 / NH1	Glu81 / OE2	4.9	--	--	--
Arg68 / NH1	Glu83 / OE1	4.8	Arg68 / NH1	Glu83 / OE1	4.3
Arg68 / NH1	Glu83 / OE2	4.1	Arg68 / NH1	Glu83 / OE2	4.6
Arg68 / NH2	Glu83 / OE1	4.4	Arg68 / NH2	Glu83 / OE1	3.9
Arg68 / NH2	Glu83 / OE2	4.4	--	--	--
--	--	--	Lys74 / NZ	Asp75 / OD1	4.8
Arg90 / NH1	Glu106 / OE1	3.2	Arg90 / NH1	Glu106 / OE1	2.9
Arg90 / NH1	Glu106 / OE2	4.7	Arg90 / NH1	Glu106 / OE2	4.7
Arg90 / NH2	Glu106 / OE1	4.6	Arg90 / NH2	Glu106 / OE1	3.7
--	--	--	Arg90 / NH2	Glu106 / OE2	4.6
Arg100 / NH1	Asp98 / OD1	4.5	--	--	--
Arg100 / NH1	Asp98 / OD2	4.6	--	--	--
--	--	--	Arg100 / NH2	Asp98 / OD1	4.5
--	--	--	Arg118 / NH1	Asp117 / OD1	3.9
Lys131 / NZ	Asp98 / OD1	2.6	Lys131 / NZ	Asp98 / OD1	3.0
Lys131 / NZ	Aps98 / OD2	3.9	Lys131 / NZ	Asp98 / OD2	4.4
Arg151 / NH1	Asp168 / OD1	4.1	--	--	--
--	--	--	Arg151 / NH1	Asp168 / OD2	3.5
Arg151 / NH2	Asp168 / OD1	4.4	--	--	--
--	--	--	Arg151 / NH2	Asp168 / OD2	3.9
Arg158 / NH1	Asp166 / OD1	4.8	--	--	--
Arg158 / NH1	Asp166 / OD2	4.6	--	--	--
Arg158 / NH2	Asp166 / OD1	3.3	--	--	--
Arg158 / NH2	Asp166 / OD2	2.7	--	--	--
Arg167 / NH1	Asp174 / OD1	2.2	Arg167 / NH1	Asp174 / OD1	3.6
Arg167 / NH1	Asp174 / OD2	3.4	Arg167 / NH1	Asp174 / OD2	3.7
Arg167 / NH2	Asp174 / OD1	3.9	--	--	--
Arg167 / NH2	Asp174 / OD2	3.7	--	--	--
--	--	--	Arg167 / NH1	Asp290 / OD1	**2.7**

--	--	--	Arg167 / NH1	Asp290 / OD2	**3.2**
Arg167 / NH2	Asp290 / OD1	3.2	Arg167 / NH2	Asp290 / OD1	**3.2**
Arg167 / NH2	Asp290 / OD2	3.4	Arg167 / NH2	Asp290 / OD2	**4.6**
Arg194 / NH1	Asp161 / OD1	3.4	Arg194 / NH1	Asp161 / OD1	2.6
Arg194 / NH1	Asp161 / OD2	2.9	Arg194 / NH1	Asp161 / OD2	4.4
Arg194 / NH2	Asp161 / OD1	2.8	Arg194 / NH2	Asp161 / OD1	3.6
Arg194 / NH2	Asp161 / OD2	3.8	--	--	--
Arg194 / NH1	Asp192 / OD2	4.5	Arg194 / NH1	Asp192 / OD2	4.4
Arg194 / NH2	Asp192 / OD1	4.5	Arg194 / NH2	Asp192 / OD1	4.6
Arg194 / NH2	Asp192 / OD2	2.8	Arg194 / NH2	Asp192 / OD2	2.7
--	--	--	Arg197 / NH1	Asp199 / OD1	3.9
--	--	--	Arg197 / NH1	Asp199 / OD2	3.0
Arg197 / NH2	Asp199 / OD1	3.7	--	--	--
Arg197 / NH2	Asp199 / OD2	3.2	Arg197 / NH2	Asp199 / OD2	4.9
--	--	--	Arg197 / NH1	Glu207 / OE1	3.3
Arg197 / NH1	Glu207 / OE2	3.4	Arg197 / NH1	Glu207 / OE2	4.2
Arg197 / NH2	Glu207 / OE2	4.8	--	--	--
Arg201 / NH1	Asp199 / OD2	4.1	--	--	--
Arg201 / NH2	Asp199 / OD1	4.2	--	--	--
Arg201 / NH2	Asp199 / OD2	3.2	Arg201 / NH2	Asp199 / OD2	4.9
--	--	--	Arg205 / NH2	Glu207 / OE1	3.8
Arg217 / NH1	Glu215 / OE2	3.7	--	--	--
Arg217 / NH2	Glu215 / OE2	4.8	--	--	--
Arg224 / NH1	Asp97 / OD1	4.7	Arg224 / NH1	Asp97 / OD1	4.4
Arg224 / NH1	Asp97 / OD2	2.7	Arg224 / NH1	Asp97 / OD2	3.5
Arg224 / NH2	Asp97 / OD1	4.6	--	--	--
Arg224 / NH2	Asp97 / OD2	3.2	--	--	--
--	--	--	Arg229 / NH1	Asp225 / OD1	4.9
--	--	--	Arg229 / NH1	Asp225 / OD2	4.5
--	--	--	Arg229 / NH2	Asp225 / OD1	4.4
--	--	--	Arg229 / NH2	Asp225 / OD2	3.0
--	--	--	Arg229 / NH1	Glu227 / OE1	4.9
Arg271 / NH1	Asp286 / OD1	4.5	Arg271 / NH1	Asp286 / OD1	4.9
Arg271 / NH2	Asp286 / OD1	3.3	Arg271 / NH2	Asp286 / OD1	2.6
Arg271 / NH2	Asp286 / OD2	5.0	Arg271 / NH2	Asp286 / OD2	3.9
Arg276 / NH1	Glu287 / OE1	4.0	Arg276 / NH1	Glu287 / OE1	3.5
Arg276 / NH2	Glu287 / OE1	4.0	Arg276 / NH2	Glu287 / OE1	2.6
--	--	--	Arg276 / NH2	Glu287 / OE2	4.7
Arg276 / NH1	Glu292 / OE1	4.9	Arg276 / NH1	Glu292 / OE1	4.9
Arg276 / NH2	Glu292 / OE1	2.8	Arg276 / NH2	Glu292 / OE1	3.6

Arg276 / NH2	Glu292 / OE2	3.2	Arg276 / NH2	Glu292 / OE2	3.2
--	--	--	Arg300 / NH1	Glu8 / OE1	3.4
--	--	--	Arg300 / NH2	Glu8 / OE1	4.3
Lys303 / NZ	Asp457 / OD1	4.0	Lys303 / NZ	Asp457 / OD1	3.8
--	--	--	Lys303 / NZ	Asp457 / OD2	5.0
Lys304 / NZ	Asp337 / OD1	3.0	Lys304 / NZ	Asp337 / OD1	3.5
Lys304 / NZ	Asp337 / OD2	2.8	Lys304 / NZ	Asp337 / OD2	3.5
Arg308 / NH1	Glu297 / OE2	4.6	Arg308 / NH1	Glu297 / OE2	4.9
Lys330 / NZ	Glu297 / OE1	2.8	Lys330 / NZ	Glu297 / OE1	2.7
Lys330 / NZ	Glu297 / OE2	4.9	Lys330 / NZ	Glu297 / OE2	4.6
Arg341 / NH1	Glu339 / OE1[A]	3.0	--	--	--
Arg341 / NH1	Glu339 / OE2[A]	3.5	Arg341 / NH1	Glu339 / OE2	4.6
--	--	--	Arg341 / NH2	Glu339 / OE1	4.2
--	--	--	Arg341 / NH2	Glu339 / OE2	2.7
--	--	--	Arg343 / NH2	Asp420 / OD2	4.0
--	--	--	Arg343 / NH1	Asp450 / OD1	4.6
Arg343 / NH1	Asp450 / OD2	4.9	Arg343 / NH1	Asp450 / OD2	2.6
Arg343 / NH2	Asp450 / OD1	4.7	--	--	--
Arg343 / NH2	Asp450 / OD2	2.6	Arg343 / NH2	Asp450 / OD2	3.9
Arg344 / NH1	Asp420 / OD1	3.0	Arg344 / NH1	Asp420 / OD1	2.9
Arg344 / NH1	Asp420 / OD2	4.1	Arg344 / NH1	Asp420 / OD2	4.3
Arg344 / NH2	Asp420 / OD1	3.5	Arg344 / NH2	Asp420 / OD1	3.0
Arg344 / NH2	Asp420 / OD2	4.4	Arg344 / NH2	Asp420 / OD2	4.5
Arg348 / NH1	Asp337 / OD1	3.8	--	--	--
--	--	--	Arg348 / NH2	Glu339 / OE1	4.6
--	--	--	Lys353 / NZ	Glu368 / OE1	4.5
--	--	--	Lys353 / NZ	Glu368 / OE2	3.4
Lys379 / NZ	Glu288 / OE1	3.0	Lys379 / NZ	Glu288 / OE1	3.0
Lys379 / NZ	Glu288 / OE2	2.6	Lys379 / NZ	Glu288 / OE2	2.9
Arg389 / NH1	Glu492 / OE2	3.7	Arg389 / NH1[A]	Glu492 / OE2	4.5
--	--	--	Arg389 / NH2[B]	Glu492 / OE2	4.2
Arg394 / NH1	Asp405 / OD1	2.7	--	--	--
--	--	--	Arg398 / NH1	Asp405 / OD1	2.9
Arg398 / NH1	Asp405 / OD2	3.1	Arg398 / NH1	Asp405 / OD2	3.0
Arg398 / NH2	Asp405 / OD1	4.3	Arg398 / NH2	Asp405 / OD1	3.2
Arg398 / NH2	Asp405 / OD2	3.4	Arg398 / NH2	Asp405 / OD2	3.9
Lys399 / NZ	Asp412 / OD1	4.3	--	---	-
--	--	--	Arg408 / NH1	Asp405 / OD2	4.9
Arg408 / NH2	Asp412 / OD1	4.4	--	--	--
Arg408 / NH2	Asp412 / OD2	3.5	--	--	--

Lys426 / NZ	Glu417 / OE1	4.5	Lys426 / NZ	Glu417 / OE1	4.4
Lys426 / NZ	Glu417 / OE2	3.3	Lys426 / NZ	Glu417 / OE2	2.8
Lys431 / NZ	Asp446 / OD1	4.5	Lys431 / NZ	Asp446 / OD1	3.4
Lys431 / NZ	Asp446 / OD2	2.6	Lys431 / NZ	Asp446 / OD2	4.8
Lys460 / NZ	Asp457 / OD2	3.7	Lys460 / NZ	Asp457 / OD2	3.1
Lys460 / NZ	Glu492 / OE2	4.8	--	--	--
Lys464 / NZ	Asp342 / OD1	2.8	Lys464 / NZ	Asp342 / OD1	3.1
Lys464 / NZ	Asp342 / OD2	4.1	Lys464 / NZ	Asp342 / OD2	4.2
Lys464 / NZ	Glu454 / OE1	4.0	Lys464 / NZ	Glu454 / OE1	4.1
Arg468 / NH1	Glu483 / OE1	4.5	Arg468 / NH1	Glu483 / OE2	4.8
Arg468 / NH1	Glu483 / OE2	3.6	Arg468 / NH1	Glu483 / OE2	3.7
Arg468 / NH2	Glu483 / OE1	3.8	--	--	--
Arg468 / NH2	Glu483 / OE2	3.9	Arg468 / NH2	Glu483 / OE2	4.9
--	--	--	Lys471 / NZ	Asp486 / OD1	4.3
--	--	--	Lys471 / NZ	Asp486 / OD2	4.0
Lys479 / NZ	Asp438 / OD2	4.6	--	--	--
Lys479 / NZ	Asp473 / OD2	3.9	Lys479 / NZ	Asp473 / OD2	3.9

For clarity, all salt bridge interactions observed in both chains A and B of PDB 3P53 are color-coded according to the corresponding β-trefoil domains in chain A of the Fascin1 structures presented in this study: magenta for β-trefoil domain 1, orange for β-trefoil domain 2, green for β-trefoil domain 3, and cyan for β-trefoil domain 4.
